# A double‐Cox model for non‐proportional hazards survival analysis with frailty

**DOI:** 10.1002/sim.9760

**Published:** 2023-05-15

**Authors:** Alexander Begun, Elena Kulinskaya, Njabulo Ncube

**Affiliations:** ^1^ School of Computing Sciences University of East Anglia Norwich UK

**Keywords:** Cox regression, marginal likelihood, parametric survival analysis, shape and scale modeling, shared frailty

## Abstract

The Cox regression, a semi‐parametric method of survival analysis, is extremely popular in biomedical applications. The proportional hazards assumption is a key requirement in the Cox model. To accommodate non‐proportional hazards, we propose to parameterize the shape parameter of the baseline hazard function using the additional, separate Cox‐regression term which depends on the vector of the covariates. This parametrization retains the general form of the hazard function over the strata and is similar to one in Devarajan and Ebrahimi (Comput Stat Data Anal. 2011;55:667–676) in the case of the Weibull distribution, but differs for other hazard functions. We call this model the double‐Cox model. We formally introduce the double‐Cox model with shared frailty and investigate, by simulation, the estimation bias and the coverage of the proposed point and interval estimation methods for the Gompertz and the Weibull baseline hazards. For real‐life applications with low frailty variance and a large number of clusters, the marginal likelihood estimation is almost unbiased and the profile likelihood‐based confidence intervals provide good coverage for all model parameters. We also compare the results from the over‐parametrized double‐Cox model to those from the standard Cox model with frailty in the case of the scale‐only proportional hazards. The model is illustrated on an example of the survival after a diagnosis of type 2 diabetes mellitus. The R programs for fitting the double‐Cox model are available on Github.

## 1 INTRODUCTION

Survival analysis is widely used in modeling time‐to‐failure data. Both semi‐parametric and parametric methods provide powerful tools for estimating the underlying hazard functions and effects of the covariates. The Cox regression,^1^ a semi‐parametric method, is almost a default method of survival analysis in biomedical applications.

The semi‐parametric approach is based on the maximum likelihood Breslow estimators of the cumulative baseline hazard function where this function is regarded as infinite‐dimensional parameter and the maximum partial likelihood estimators for the Cox‐regression parameters.^2^ The proportional hazards assumption is a key requirement in the Cox model. In the parametric approach, the baseline hazard function has a parametric form corresponding to the exponential, Weibull, Gompertz or other survival distribution. Although the parametric approach guarantees more power than the semi‐parametric one if the form of the baseline hazard function is known, the semi‐parametric method is preferable if it is not the case.

Proportional hazard model under frailty setting is a natural extension of the simple proportional hazards model. A non‐negative random variable Z (frailty) is added to the model, so that the instantaneous conditional risk of failure at the moment t, $h(t|u,Z)=Z\chi (u)h_{0}(t)$ is proportional to the baseline hazard function $h_{0}(t)$, the non‐negative function χ(u) for the vector of observed covariates u, and a value of Z. The function χ(u) is usually defined as exp(βu) (the Cox‐regression term) and specifies the fixed effects. The frailty Z is included to take into account the influence of unobserved factors and to avoid specification of the joint distributions for dependent failures (eg, deaths of individuals belonging to the same family or to the same cluster).

However, for real‐world data, the proportional hazards assumption may not be satisfied. A most general, extended Cox regression would include both time‐varying covariates and regression effects. For analysis of extended multiplicative hazards models see Martinussen and Scheike.^3^ ^(Chapter 6)^ Stratification is a useful and a much simpler option for non‐proportional hazards analysis, allowing to take into account different forms of the baseline hazard functions in different strata. This option is widely available in statistical software relating to survival analysis. In the parametrical approach, the baseline hazard functions in different strata need to be specified parametrically. Yashin et al.^4^ discussed four methods of specifying the survival functions for different genotypes in the longevity studies. These are: the nonparametric, the relative risk, the parametric and the semi‐parametric approaches. Begun^5^ used the power transform $H_{0}{\left(t\right)}^{p_{G}}$ with a nonnegative power $p_{G}$ to determine the cumulative hazard function for individuals with genotype G. Devarajan and Ebrahimi^6^ proposed the non‐proportional hazard regression model, where the baseline cumulative hazard function is raised to a power depending on the covariates: $H_{0}{\left(t\right)}^{\exp(\zeta u)}$.

In this article, we consider an alternative method of specifying the hazard functions in different strata. Namely, we assume that the shape parameter of the baseline hazard function can be specified using the additional, separate Cox‐regression term. That is, the shape parameter b(u) of the hazard function is written as $b\exp(\beta_{b}u)$ and depends on the vector of the covariates. This parametrization retains the general form of the hazard function over the strata and is similar to one in Devarajan and Ebrahimi^6^ in the case of the Weibull distribution, but differs for other hazard functions. We call this model a double‐Cox model. The choice of name reflects the presence of dual Cox‐regression terms, to allow modeling of both scale and shape.

This form of parametrization appears to be more interpretable than that of Devarajan and Ebrahimi,^6^ since the form of the baseline hazard in each stratum can be meaningful. For example, in the Strehler‐Mildvan model,^7^ the shape parameter b of the Gompertz hazard function is proportional to the rate of vitality decline. As an another example, Abernethy^8^ showed that exponential increase of the mortality rate can be caused by exponential increase in time of the mitotic event waiting‐time.

The double‐Cox model with shared frailty was successfully applied to analysis of time‐to‐failure of hip replacements^9^ and to analysis of effects of HRT^10^ and of a diagnosis of type‐2 diabetes mellitus^11^ on longevity. In present paper, we formally introduce the model and investigate, by simulation, estimation bias and the coverage of the proposed estimation methods. In Section 2, we discuss point and interval parameter estimation. Simulation design is described in Section 3. The results of the simulations are described in Section 4. We also compare the results from the over‐parametrized double‐Cox model to those from the standard Cox model and from the *parfm* R package for parametric frailty models in the case of the scale‐only proportional hazards.^12^ Section 5 provides an example of the survival after a diagnosis of type 2 diabetes mellitus. Summary and discussion are in Section 6. The R programs for fitting the double‐Cox model are available on Github, see https://rdrr.io/github/AB5103/doubleCoxr/ for details.

## 2 MODEL AND ML ESTIMATION

### 2.1 The double‐Cox model with shared frailty

Consider a non‐proportional hazard regression model with frailty Z and a specified two‐parameter (scale and shape) baseline hazard function, where both parameters log‐linearly depend on the observed covariates. In this study, we consider two baseline distributions, the Gompertz and the Weibull. The Gompertz hazards are typically used in human longevity studies and the Weibull hazards in modeling time‐to‐failure of technical devices. The cumulative conditional hazard function is defined as

(1) $$\tilde{H}(t|u,Z)=ZH(t|u)=Ze^{\beta_{\text{scale}}u}{\left(\frac{t}{a}\right)}^{b\exp(\beta_{\text{shape}}u)}$$

in the case of the Weibull model and as

(2) $$\tilde{H}(t|u,Z)=ZH(t|u)=Zae^{\beta_{\text{scale}}u}\frac{\left(e^{b\exp(\beta_{\text{shape}}u)t}-1\right)}{b\exp(\beta_{\text{shape}}u)}$$

in the case of the Gompertz model. Here a>0 and b>0 are the scale and the shape parameters of the baseline survival distribution, respectively, u is the vector‐column of the covariates, $\beta_{\text{scale}}$ and $\beta_{\text{shape}}$ are the vector‐rows of the Cox‐regression parameters.

We consider the gamma‐distributed frailty Z with mean 1, variance $\sigma^{2}$ and probability density function $f(z|\sigma^{2})$ and define the “random effect” by ω=lnZ. We also assume that the population is divided into $N_{cl}$ clusters and all $n_{i}$ individuals from the cluster i, $i=1,\ldots ,N_{cl}$, share the same frailty $Z_{i}$. The total sample size $N=\sum_{i=1}^{N_{cl}}n_{i}$.

The conditional and the marginal survival functions are given by

$$\begin{array}{llll} S(t|u,Z) & =\exp(-\tilde{H}(t|u,Z)),\; & & \\ S(t|u) & =𝔼S(t|u,Z)={\left(1+\sigma^{2}H(t|u)\right)}^{-1/\sigma^{2}}.\; & & \end{array}$$

The conditional likelihood function is defined by

(3) $${ℒ}_{c}(\text{Data}|\zeta ,Z_{1},\ldots ,Z_{N_{cl}})=\overset{N_{cl}}{\underset{i=1}{\prod }}\overset{n_{i}}{\underset{j=1}{\prod }}{\left(-\frac{\partial }{\partial t_{ij}}\right)}^{\delta_{ij}}\exp(-\underset{j}{\sum }\tilde{H}(t_{ij}|u_{ij},Z_{i})),$$

where $\zeta =(a,b,\beta_{\text{scale}},\beta_{\text{shape}})$ is the vector of parameters, $\delta_{ij}$ stands for censoring (1 if censored and 0, otherwise) and indices i and j correspond to a cluster and a subject in that cluster, respectively. We consider parameter estimation in the model (3) in the next section.

### 2.2 Point estimation of the parameters

Marginal likelihood is calculated by marginalizing out the frailty Z in the conditional likelihood (3). From a random effect, the marginal likelihood inherits only parameter $\sigma^{2}$. The k‐dimensional vector of the unknown parameters $\xi =(\zeta ,\sigma^{2})$ is estimated by maximizing the marginal likelihood function (or its logarithm)

(4) $${ℒ}_{m}(\text{Data}|\xi )=𝔼{ℒ}_{c}(\text{Data}|\zeta ,Z_{1},\ldots ,Z_{N_{cl}})=\underset{i,j}{\prod }{\left(-\frac{\partial }{\partial t_{ij}}\right)}^{\delta_{ij}}{\left(1+\sigma^{2}\underset{j}{\sum }H(t_{ij}|u_{ij})\right)}^{-1/\sigma^{2}}.$$

The marginal likelihood function (4) is a Laplace transform of the frailty distribution calculated at the point H(t|u). The Gamma frailty results in a closed‐form marginal likelihood.

Unfortunately, not all frailty distributions have their Laplace transform in a simple closed form. The log‐normal frailty distribution often used in survival analysis is an example of such an “inconvenient” distribution.^13^ For a general frailty, the parameters of the survival model could be estimated using intensive procedures of numerical integration. The expectation‐maximization (EM) algorithm for computing parameter estimates is an alternative approach to obtain the ML estimates.^14^ We provide the requisite steps of the EM algorithm in Supplementary Material A.1. Slow convergence and intensive calculations in comparison to the estimation based on the maximization of the closed‐form marginal likelihood is the price for using the EM‐algorithm.

The penalized likelihood approach is an alternative to the EM‐algorithm to obtain the ML estimates in the presence of the unobserved random effects. In the case of gamma‐distributed frailties this approach results in the same parameter estimates as the EM‐algorithm and involves iterative estimation of the random effects (log‐frailties). The idea of the method is to exclude unlikely small or unlikely large values of the frailty by subtracting a penalty function from the conditional likelihood. The solution to the penalized log‐likelihood coincides with the EM solution for any fixed value of $\sigma^{2}$.^15^, ^16^ Furthermore, the estimate of $\sigma^{2}$ can be found through maximizing the profile likelihood. See Supplementary Material A.1 for more details.

### 2.3 Confidence intervals for the parameters

#### 2.3.1 Standard‐error based confidence intervals

Information about the standard errors of the parameter estimates can be obtained from the inverse of the Hessian matrix. Let ξ be the k‐vector parameter and $\hat{\xi }$ its maximum likelihood estimate (MLE). Assume that $\left[\xi_{j}^{l},\xi_{j}^{u}\right]$ is the 1−α confidence interval for the jth component ${\hat{\xi }}_{j}$, j=1,…,k. The coverage probability for component j is defined by

$$P_{cov}(\xi_{j})=\mathbb{P}(\xi_{j}\in [\xi_{j}^{l},\xi_{j}^{u}]).$$

This probability can be estimated from simulations as a proportion of cases when the true value of $\xi_{j}$ lies in interval $\left[\xi_{j}^{l},\xi_{j}^{u}\right]$.

The scale and the shape parameters a and b of the baseline hazard distribution and the Cox‐regression parameters $\beta_{\text{scale}}$ and $\beta_{\text{shape}}$ do not lie on the boundary. But the frailty variance $\sigma^{2}$ can be equal to zero corresponding to the model without frailty. If $\sigma^{2}>0$ and $\sigma^{2}$ is large compared with the standard error for ${\hat{\sigma }}^{2}$, the confidence intervals can be specified using the asymptotic normality of MLE:

$$\sqrt{N}(\hat{\xi }-\xi )\overset{d}{\underset{\;}{\to }}𝒩(0,\sum ),$$

where N is the number of observations, 𝒩 is the k‐variate normal distribution with zero mean and the k×k asymptotical variance‐covariance matrix ∑ with elements $\sum_{l,m}$, l,m=1,…,k. In general, for any values of $\sigma^{2}$, the confidence intervals can be calculated using a mixture of the truncated normal distribution and a point mass at zero for ${\hat{\sigma }}^{2}$, as in Böhnstedt and Gampe^17^:

(5) $$\begin{array}{ll} \sqrt{N}({\hat{\xi }}_{j}-\xi_{j}) & \overset{d}{\underset{\;}{\to }}\Phi \left(\frac{\nu }{\kappa }\right){𝒯𝒩}_{1}(\mu_{\nu },\sum_{j},s,t)+\Phi \left(-\frac{\nu }{\kappa }\right)𝒩\left(-\frac{\sum_{j,k}}{\sum_{k,k}}\nu ,\sum_{k,k}-\frac{\sum_{j,k}^{2}}{\sum_{k,k}}\right),\\ \sqrt{N}{\hat{\sigma }}^{2} & \overset{d}{\underset{\;}{\to }}\Phi \left(\frac{\nu }{\kappa }\right)𝒯𝒩(\nu ,\kappa^{2},0,\infty )+\Phi \left(-\frac{\nu }{\kappa }\right)\chi_{0}^{2}, \end{array}$$

where $\chi_{0}^{2}$ is a point mass at zero, $\kappa =\sqrt{\sum_{k,k}}$, $\nu =\sqrt{N}\sigma^{2}$, $\mu_{\nu }={\left(0,\nu \right)}^{T}$, $\sum_{j}=\left(\begin{array}{cc} \sum_{j,j} & \sum_{j,k}\\ \sum_{k,j} & \sum_{k,k} \end{array}\right)$, j=1,…,k−1, 𝒯𝒩(x,V,0,∞) and ${𝒯𝒩}_{1}(x,V,s,t)$ are the truncated to (0,∞) univariate normal distribution 𝒩(x,V) and the marginal distribution of the first component of a truncated bivariate normal distribution ${𝒩}_{2}(x,V)$ with the lower and upper truncation limits $s={\left(-\infty ,0\right)}^{T}$ and $t={\left(\infty ,\infty \right)}^{T}$, respectively. We call these intervals standard‐error‐based.

#### 2.3.2 Profile likelihood based confidence intervals

The standard‐error based confidence intervals may have low coverage. The profile likelihood (PL) based confidence intervals are an attractive alternative. The profile likelihood method inverts the likelihood ratio test to obtain a confidence interval for the parameter under study. Let (ξ,ψ) be a (p+q)‐dimensional vector of the p unknown parameters of interest ξ and the q nuisance parameters ψ in a statistical model. Denote by Lik(ξ,ψ) the likelihood function, and by $\left(\xi^{*},\psi^{*}\right)$ the maximum likelihood estimates, $\left(\xi^{*},\psi^{*})=\arg\;\max_{\xi ,\psi }Lik(\xi ,\psi \right)$. Define the profile likelihood function $Lik_{p}(\xi )$ by maximizing the function Lik(ξ,ψ) over the parameter ψ, that is, $Lik_{p}(\xi )=\max_{\psi }Lik(\xi ,\psi )$. A point ξ lies in the 95% confidence interval for $\xi_{0}$ (true p‐dimensional parameter of interest) if $2(\ln Lik_{p}(\xi^{*})-\ln Lik_{p}(\xi_{0}))\leq \chi_{.95}^{2}(p)$, where $\chi_{.95}^{2}(p)$ is the 95th percentile of the $\chi^{2}$ distribution with p degrees of freedom. For example, if the parameter of interest is the frailty variance then p=1 and $\chi_{.95}^{2}(1)=3.84$. The coverage probability of the 95% confidence interval for parameter ξ can be estimated from simulations as a proportion of cases when the true value $\xi_{0}$ lies in the nominal 95% interval.

## 3 DESIGN OF THE SIMULATIONS

In the simulation study, we investigate the quality of the parameter estimation for a double‐Cox model with two covariates: a binary “Success” and a continuous “Score,” for both the Gompertz and the Weibull baseline hazards. The scale and shape parameters of these distributions were fixed at a=20, b=1.5 for Weibull and at a=0.0001, b=0.1 for Gompertz hazards. The variance of the gamma‐distributed frailty $\sigma^{2}$ varied from zero to 5. The choice of the shape and scale parameters guaranteed a realistic life expectancy. As an example, when the frailty variance $\sigma^{2}=1$, the life expectancy for a Gompertz survival model with the above parameters is equal to 69.2. For the Weibull survival model with the above parameters and frailty variance 1, the life expectancy is 17.8. These life expectancies are comparable to human longevity and the longevity of a hip prosthesis, respectively.^9^

The binary covariate “Success” was generated from the Bernoulli($p_{\text{Success}}$) distribution for each individual in a study. The covariate “Score” was generated from the normal distribution with mean 0 and variance 0.2 independently of the covariate “Success.” The corresponding Cox‐regression vector‐parameters $\beta_{\text{shape}}$ and $\beta_{\text{scale}}$ were chosen independently of each other. To make effects of scale and shape parameters comparable, the Cox‐regression coefficients for shape parameters were chosen to be one order smaller than those for scale.

Apart of the parameters of the model a, b, $\beta_{\text{scale}}$, $\beta_{\text{shape}}$, and $\sigma^{2}$, we also studied the effects of the additional structure parameters of the data such as the total sample size N, the number of clusters $N_{cl}$, the proportion $p_{\text{Success}}$ of the baseline values for the binary covariate and the censoring rate $p_{\text{cens}}$. 5000 repetitions were used for each of 1440 configurations of the parameters. The full list of the parameters used in simulations is provided in Table 1.

**TABLE 1:** Values of parameters in the simulations.

Parameter	Values
Number of repetitions	5000
Sample size N	300^*^, 1000^*^, 10 000
Number of clusters $N_{cl}$	10^*^, 100^*^
Censoring rate $p_{\text{cens}}$	0^*^, 0.4^*^, 0.8
Proportion $p_{\text{Success}}$ of 1's in the variable “Success”	0.25, 0.5^*^
$\left(\beta_{\text{scale‐success}},\beta_{\text{scale‐score}}\right)$	(0.5, 1), (−0.5, ‐1)^*^
$\left(\beta_{\text{shape‐success}},\beta_{\text{shape‐score}}\right)$	(0.05, 0.1), (−0.05, −0.1)
True variance of frailty $\sigma^{2}$	0^*^, 0.5, 1, 2^*^, 5
($a_{\text{Gompertz}},b_{\text{Gompertz}}$)	(0.0001^*^, 0.1^*^)
($a_{\text{Weibull}},b_{\text{Weibull}}$)	(20^*^, 1.5^*^)
“Success”	*Bernoulli(* $p_{\text{Success}}$ *)*
$p_{\text{Success}}$	0.25, 0.5
“Score”	𝒩(0,0.2)

* Parameters of the additional simulations for the over‐parametrized model.

Additionally, to test the behavior of the parameter estimates for an over‐parametrized double‐Cox model with zero shape, we generated the data using a subset of the parameters, resulting in 16 configurations also listed in Table 1.

R statistical software^18^ was used for simulations. To fit the models, we used our R package *doubleCoxr* available at https://rdrr.io/github/AB5103/doubleCoxr/ and the “parfm”^12^ function was used for comparative purposes when fitting the over‐parametrized models.

The details of specifying a simulation with a predefined proportion of censoring are described in Supplementary Material A.2. In brief, similar to Wan,^19^ the censoring times $c_{i}$ for individuals i=1,…,N, are generated from Uni(0,θ) distribution. Given the population censoring rate $p_{\text{cens}}$, the unknown parameter θ is a numerical solution to the equation $\mathbb{P}(\delta =1|\theta )=p_{\text{cens}}$. In our simulations, parameter θ was estimated from Equations (A.1) and (A.2) in Supplementary Material A.2 with $N_{\text{sim}}=10^{6}$ Monte‐Carlo simulations.

The estimates of unknown parameters $\left(a,b,\beta_{\text{scale}},\beta_{\text{shape}},\sigma^{2}\right)$ were calculated maximizing the marginal likelihood for 5000 simulations and then averaged. We studied the bias of estimation for all parameters, and also coverage of the standard‐error‐based and the profile‐likelihood‐based confidence intervals at nominal 95% level.

## 4 SIMULATION RESULTS

Our full simulation results are available as e‐print.^20^ Here we provide a summary illustrated by some typical figures.

### 4.1 Biases in the parameter estimation

**FIGURE 1:**
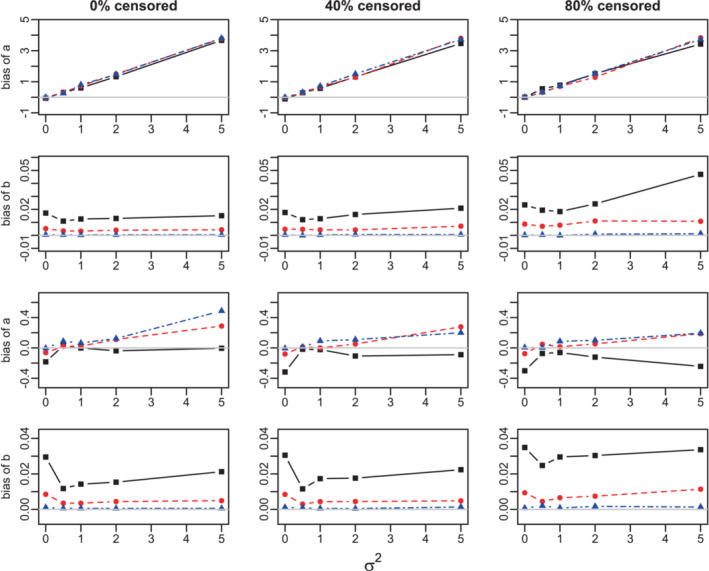
Bias of the estimation of a and b parameters in the Weibull model. Success proportion= 0.25. Sample sizes: 300 (black solid lines, squares), 1000 (red dashed lines, circles), and 10 000 (blue dot‐dashed lines, triangles). True values: $\beta_{success-scale}=0.5$, $\beta_{\text{success‐shape}}=0.05$, $\beta_{\text{score‐scale}}=1$, $\beta_{\text{score‐shape}}=0.1$. Top two rows: 10 clusters; bottom two rows: 100 clusters.

The estimation of the parameters “a” and “b” of the baseline distribution is almost unbiased for small values of the frailty variance $\sigma^{2}$. However, the bias in estimation of a grows linearly with $\sigma^{2}$ and may be considerable when $N_{cl}=10$. This bias is positive for the Weibull, and negative for the Gompertz distribution. The bias in estimation of both a and b decreases in the number of clusters. Figures 1, A.1–A.3.

The estimation of the Cox regression scale parameters is almost unbiased for censoring rates up to 40%, but is noticeably biased when the high censoring rate of 80% is combined with a modest sample size. For N=300, the estimates of the shape parameters, are considerably more biased than their scale counterparts, especially when $\sigma^{2}\leq 2$ or for high censoring rate. The sign of the bias depends on the sign of the coefficients, Figures A.4 and A.5 (10 clusters).

Smaller censoring rate increases the number of informative cases. In a sense, this effect is equivalent to an increase in the sample size. It seems that 200 of informative cases (corresponding to the sample size of 1000 and censoring rate of 0.8) are sufficient for a relatively good estimation of the Cox‐regression parameters with bias not exceeding 10% of their true values.

Although the model is identifiable, the shape and the scale parameters relating to the same covariates can compete for the likelihood in searching for the maximum likelihood estimates. This can slow down the convergence of the estimates to their true values.

**FIGURE 2:**
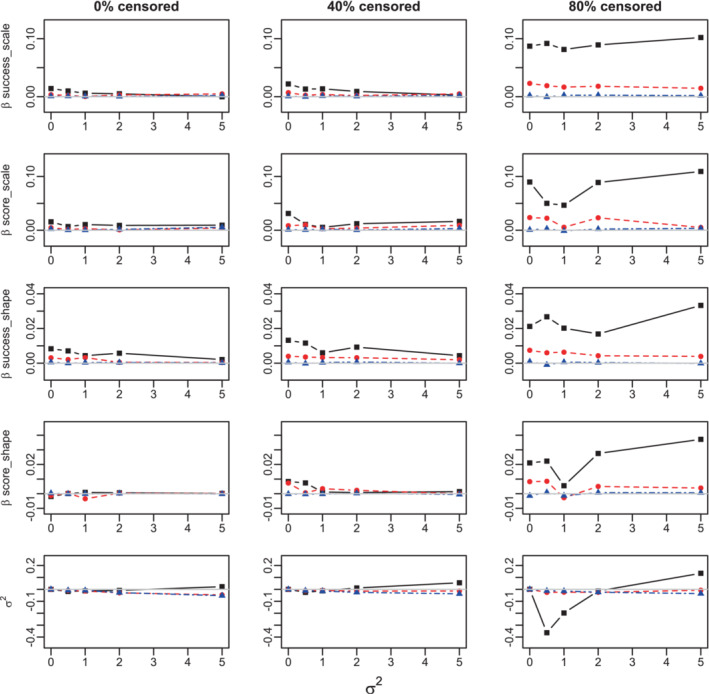
Bias of the estimation of the Cox regression parameters and $\sigma^{2}$ in the Weibull model. Success proportion= 0.25. 100 clusters. Sample sizes: 300 (black solid lines, squares), 1000 (red dashed lines, circles), and 10 000 (blue dot‐dashed lines, triangles). True values: $\beta_{success-scale}=0.5,\beta_{\text{success‐shape}}=0.05$, $\beta_{\text{score‐scale}}=1,\beta_{\text{score‐shape}}=0.1$.

The estimation bias of ${\hat{\sigma }}^{2}$ is negative and declines practically linearly with the true value of $\sigma^{2}$, thus the relative error appears to be constant for larger sample sizes. When the number of clusters $N_{cl}=100$, the bias of the Cox‐regression parameters is not affected, but that of $\sigma^{2}$ is considerably reduced, Figures 2 and A.6. This effect may be due to an increase in the number of underlying frailty values equal to the number of different clusters.

Overall, these findings are in agreement with the consistency of the ML estimates and well known underestimation of $\sigma^{2}$. The results are similar for the Gompertz model, Figures A.7–A.10.

### 4.2 Coverage of the standard‐error based (SE) confidence intervals for the Cox regression parameters

SE confidence intervals (5) provide an asymptotically nominal coverage for the scale Cox‐regression parameters, but not the shape parameters, Figures A.11 and A.14. The coverage of the scale parameters suffers under high censoring combined with smaller sample sizes, and can be as low as 70%‐80% for both the Weibull and the Gompertz models. Worryingly, for some parameter combinations, the coverage of the shape parameters deteriorates with larger sample sizes, suggesting the use of a wrong limit distribution. This is likely to be related to boundary effects in estimation of $\sigma^{2}$. Coverage of all parameters improves with the number of clusters, Figures A.12 and A.15.

When $N_{cl}=10$, the coverage of the frailty variance $\sigma^{2}$ can be as low as 80% for some values of $\sigma^{2}$, Figures A.11 and A.14. Coverage improves but may still be erratic when $N_{cl}=100$, Figures A.12 and A.15. Overall, we do not recommend the use of the SE confidence intervals.

### 4.3 Coverage of the profile‐likelihood‐based (PL) confidence intervals

PL confidence intervals provide a reasonable amount of coverage to all scale and shape Cox‐regression parameters in both Weibull and Gompertz models. The coverage generally decreases with higher censoring, but it still remains well above 90% for all studied parameter combinations at a 95% nominal confidence level, when the sample size N=300 and the censoring is as high as 80%, Figures 4, A.13, A.16, and A.17. The coverage improves for lower censoring rates and converges to the nominal level for larger sample sizes.

**FIGURE 3:**
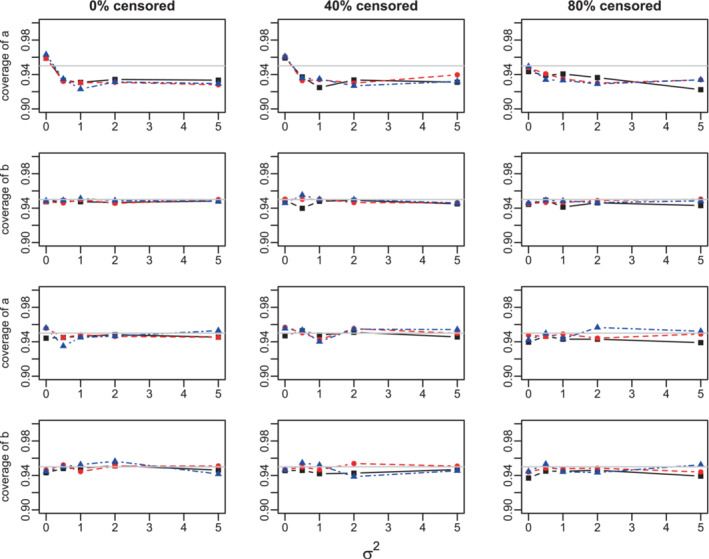
Coverage of the profile likelihood confidence intervals for a and b parameters at nominal 95% level. Weibull model. Success proportion = 0.25. Sample sizes: 300 (black solid lines, squares), 1000 (red dashed lines, circles), and 10 000 (blue dot‐dashed lines, triangles). True values: $\beta_{success-scale}=0.5,\beta_{\text{success‐shape}}=0.05,\beta_{\text{score‐scale}}=1,\beta_{\text{score‐shape}}=0.1$. Top two rows: 10 clusters; bottom two rows: 100 clusters.

The coverage of the baseline distribution parameters a and b is also acceptable. For 10 clusters, the coverage of a is somewhat below nominal, at about 92%, but it reaches nominal level for 100 clusters, Figures 3 and A.18.

**FIGURE 4:**
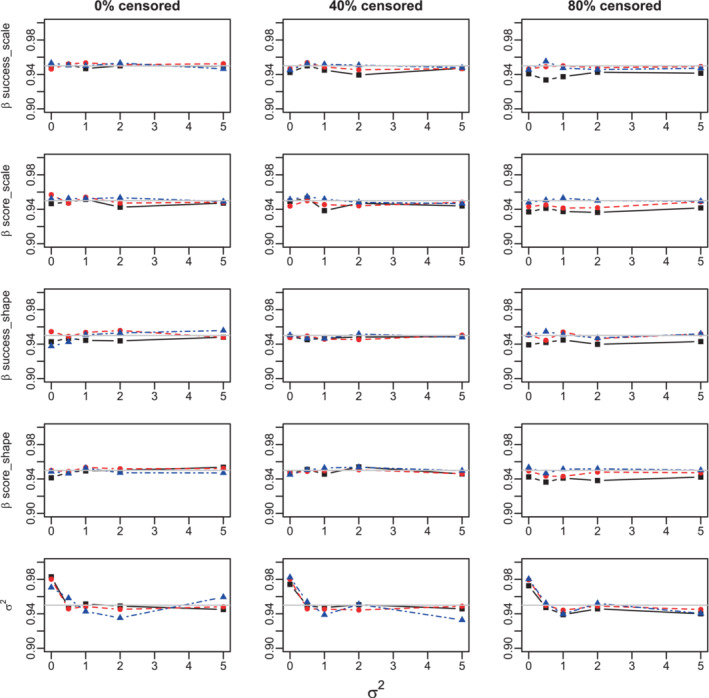
Coverage of the profile likelihood confidence intervals for the Cox regression parameters and $\sigma^{2}$ at nominal 95% level. Weibull model. Success proportion = 0.25. 100 clusters. Sample sizes: 300 (black solid lines, squares), 1000 (red dashed lines, circles), and 10 000 (blue dot‐dashed lines, triangles). True values: $\beta_{success-scale}=0.5,\beta_{\text{success‐shape}}=0.05,\beta_{\text{score‐scale}}=1,\beta_{\text{score‐shape}}=0.1$.

The coverage of $\sigma^{2}$ is usually above nominal when $\sigma^{2}=0$, and at about 92% when $\sigma^{2}>0$ even for large sample sizes when the number of clusters is 10, Figures A.13 and A.16. This parallels the negative bias in estimation of $\sigma^{2}$. The coverage improves for larger number of clusters, and is almost nominal for all parameters when $N_{cl}=100$, Figures 4 and A.17.

Overall, the PL method appears to be more robust and reliable and is recommended for use in practice.

### 4.4 Effects of over‐parametrization

To compare the quality of estimation in the over‐parametrized double‐Cox model (shape and scale parameters) with that of the true single‐Cox model (scale only), we calculated two sets of the scale estimates for the true model: using our *doubleCoxr* software for the ML estimation under the single Cox model, and using the function “parfm” from the R‐package of the same name. We also used our *doubleCoxr* function to estimate parameters for the over‐parametrized model with additional Cox‐regression term for shape b (double Cox). The results are given in Tables A.1 and A.2 in the Supplementary Materials.

The scale estimates from our single‐Cox program and the “parfm”^12^ were practically identical for all combinations of the parameters $\left(N,\;N_{cl},\;p_{\text{cens}},\;p_{\text{Success}},\;\sigma_{true}^{2}\right)$. For the binary covariate, relative error of estimation was within 2% for N=300 and within 1% for N=1000. For the continuous covariate, the relative error of estimation was within 5% for N=300 and within 1% for N=1000. The double‐Cox estimation of scale had a slightly higher relative error, within 3% for N=300, for the binary covariate, and practically the same error as the single‐Cox for N=1000 and for the continuous covariate. Therefore, the estimates are robust against over‐parametrization in the shape term.

## 5 EXAMPLE: SURVIVAL AFTER A DIAGNOSIS OF TYPE 2 DIABETES MELLITUS

In our recent study,^11^ we investigated long‐term hazards of all‐cause mortality following a diagnosis of type 2 diabetes mellitus (T2DM) using a UK electronic primary care database The Health Improvement Network (THIN). The THIN data are obtained from 711 registered general practices (GPs) and represent about 6% of the UK population.^21^

We designed a retrospective matched cohort study which included individuals born between 1930 and 1960, diagnosed with T2DM between 2000 and 2016 and aged 50‐74 years at diagnosis. Individuals with pre‐existing life‐limiting conditions and typical T2DM complications such as cancer, stroke, cognitive impairment and lower limb amputation, were excluded. The average age at diagnosis of T2DM was slightly above 61 years across the entry period. Patients with T2DM were matched at diagnosis to at most 3 controls by age, gender and GP and followed up to 1 January 2017. A total of 221 182 individuals, 30.8% with T2DM diagnosis, were selected for the study, of whom 29 618 (13.4%) died during follow‐up. The study was approved by THIN Scientific Review Committee (SRC) with approval number 16THIN095.

The proportion of study subjects aged 65 years and above was 39.4%.

Missing values in covariates were handled using the joint model multiple imputation method.^22^, ^23^ The joint modeling (JOMO) package in R was used, using the imputation model obtained from the complete‐case data.^23^ Fifteen imputed data sets were created. For inference, the results were combined using the Rubin's rules.^22^

The final survival models were adjusted for age at entry, birth cohort, gender, body‐mass index (BMI), smoking status, socio‐economic status as measured by the Townsend deprivation index (TDI), pre‐existing atrial fibrillation (AF), heart failure (HF), myocardial infarction (MI), peripheral venous disease (PVD), hypercholesteremia (HCL) and hypertension (HTN) and the following interactions: T2DM indicator with MI and smoking status, BMI with smoking and birth cohort with smoking. Tests of the proportionality assumptions and plots of scaled Schoenfeld residuals in the standard Cox model are provided in Supplementary material A5. Birth cohort (1930‐1939, 1940‐1949, 1950‐1960), HTN and AF had time‐variant effects. Comparing estimated cumulative baseline hazard function to cumulative hazards from a number of parametric survival distributions, we found that the Gompertz distribution provided a very good fit,^11^ ^(Figure S.3)^ and therefore Gompertz‐double‐Cox model with Gamma frailty on GP was used to estimate time‐varying hazards of all‐cause mortality, adjusting for medical history, socio‐demographic and lifestyle factors. See Ncube et al.^11^ for details.

Age at entry/diagnosis was categorized into two categories: 50‐59 and 60‐74 years. The baseline Gompertz parameters correspond to the baseline hazard function for T2DM‐free females aged 60‐74 years at entry, born between 1930 and 1939, from a medium deprived area (TDI = 3), non‐smokers, of normal weight, with no AF, HF, MI, PVD, HTN, and HCL. Estimated shape and scale parameters for the time‐variant covariates in the complete case model and the model obtained on the imputed data are provided in Table 2. The frailty variance was highly significant but rather low at 0.14 and 0.12 in the imputed and the complete case models, respectively. Both models have similar parameter estimates and fit the data well, with the concordance^24^, ^25^ of 75.4% and 74.8%, respectively. Other covariates were found to be time‐invariant. In brief, males had 38% increased hazard of mortality compared to females. Obesity was associated with a 1.16 [1.1‐1.23] HR while being “overweight” was not significantly different from being of normal weight. The effect of smoking on mortality increased by birth cohort. Deprivation and pre‐existing morbidity significantly increased mortality hazards.

**TABLE 2:** Scale and shape parameter estimates at baseline and for time‐variant covariates in Gompertz‐double‐Cox shared frailty model of survival after a diagnosis of T2DM for complete case and imputed data.

	Imputed			Complete case		
Parameter	Estimate	95% CI	P‐value	Estimate	95% CI	P‐value
1000a (scale)	6.73	6.24‐7.25	<1e‐16	6.64	6.07‐7.25	<1e‐16
100b (shape)	8.74	8.13‐9.39	<1e‐16	8.10	7.31‐8.98	<1e‐16
	Exponentiated covariates shape parameters					
Year of birth						
1930‐1939	1			1		
1940‐1949	0.71	0.66‐0.77	<1e‐16	0.69	0.62‐0.77	<1e‐16
1950‐1960	0.87	0.77‐0.97	0.019	0.89	0.76‐1.03	0.122
AF	1.36	1.27‐1.47	<1e‐16	1.36	1.24‐1.49	<1e‐16
HTN						
None	1			1		
Treated	1.30	1.21‐1.40	<1e‐16	1.37	1.24‐1.52	<1e‐16
Untreated	0.84	0.76‐0.94	0.0015	0.89	0.77‐1.03	0.105
	Exponentiated covariates scale parameters					
Year of birth						
1930‐1939	1			1		
1940‐1949	0.71	0.67‐0.76	<1e‐16	0.73	0.68‐0.79	<1e‐16
1950‐1960	0.47	0.43‐0.52	<0.0036	0.45	0.40‐0.50	<1e‐16
AF	0.78	0.73‐0.84	<1e‐16	0.83	0.76‐0.90	<1e‐16
HTN						
None	1			1		
Treated	0.80	0.76‐0.84	<1e‐16	0.81	0.76‐0.86	<1e‐16
Untreated	1.52	1.47‐1.6	<1e‐16	1.48	1.38‐1.58	<1e‐16
	Frailty estimate					
Variance ($\sigma^{2}$)	0.14	0.12‐0.16	<1e‐16	0.12	0.1‐0.14	<1e‐16
	Goodness of fit					
Concordance (C)	0.754			0.748		
Standard deviation of C	0.002			0.002		
Log likelihood	−145 150.22			−96 410.95		
AIC	290 386.43			192 907.9		

T2DM was associated with a HR of all‐cause mortality of 1.38 [1.32‐1.44] compared to non‐diabetics. Later diagnosis (60‐74 vs 50‐59 years of age) resulted in 25.6% higher hazards of all‐cause mortality (HR: 1.52[1.44‐1.60] vs 1.21[1.12‐1.30]). Effects of all these covariates were constant across all birth cohorts.

**FIGURE 5:**
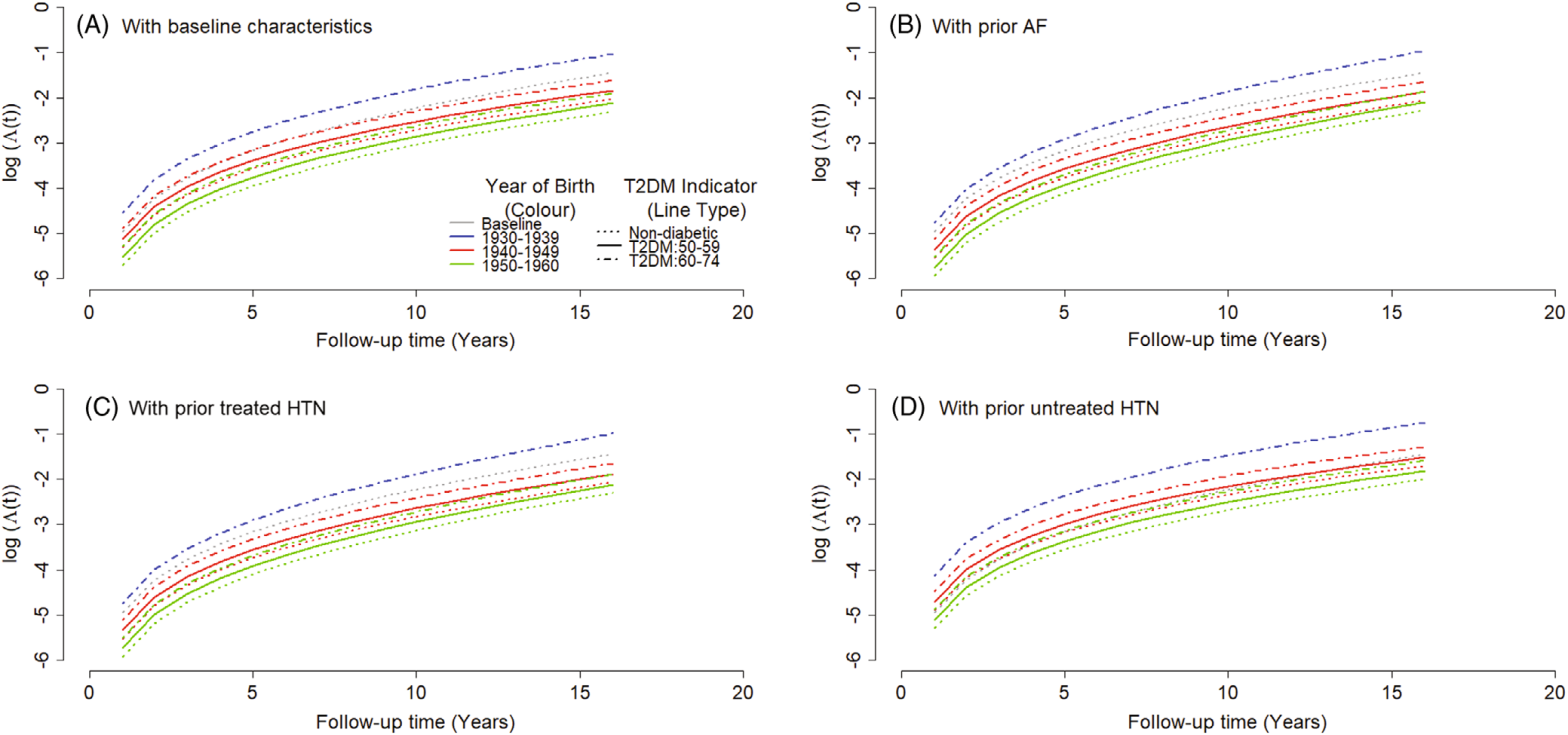
Log‐cumulative hazards of all cause mortality by birth cohort and T2DM status. The baseline Gompertz parameters correspond to the baseline hazard function for non‐diabetic females aged 60‐74 years at entry/diagnosis, born between 1930 and 1939, from a medium deprived area (TDI = 3), non‐smokers, of normal weight, with no AF, HF, MI, PVD, HTN, and HCL.

Log‐cumulative hazards of all‐cause mortality by T2DM, AF and HTN status and birth cohort are depicted in Figure 5. The 1940‐1949 and 1950‐1960 birth cohorts had considerably lower scale parameters than the 1930‐1939 birth cohort, corresponding to lower mortality hazards at study entry. The shape parameter was the lowest, at 0.71, in the 1940‐1949 cohort, but went up to 0.87 in the 1950‐1960 cohort. Accordingly, there is a considerable mortality improvement, especially at older ages, in the 1940‐1949 birth cohort in comparison to the 1930‐1939 cohort. However, there is much lower mortality improvement between the 1940‐1949 and the 1950‐1960 birth cohorts, further reduced in the older ages, irrespective of the T2DM status, Figure 5A.

Hypertension and AF increased mortality risk compared to the baseline population as shown in Figures 5B–D. Treated HTN had a much higher shape and much lower scale parameter than untreated HTN. Accordingly, Figures 5C,D show untreated HTN to be associated with increased all‐cause mortality hazards at study entry compared to people without HTN or with treated HTN. However, the all‐cause mortality risk increased among people with treated HTN, so that after 13 years, individuals with treated HTN had higher mortality hazards compared to those with untreated HTN. After 7 years of follow up, individuals with treated HTN had increased mortality hazards compared to people without HTN.

To summarize, the use of double‐Cox model allowed to investigate time‐varying effects of the birth cohort, AF and HTN. The slowing down of the mortality improvement in the younger birth cohort was an important new finding facilitated by this approach.

## 6 DISCUSSION

The semiparametric and parametric methods of survival analysis are routine and necessary tools in numerous medical, biological, and demographic studies. Parametric maximum likelihood estimation guarantees the most efficient inferential procedures when the true form of the underlying hazard function is known. To enable additional flexibility of the parametric approach, we included an additional Cox regression term modeling the shape of the baseline hazard function. This modification is easily extended to a situation when different strata of the survival data are described by different non‐proportional baseline hazard functions. In principle, any parameter of the baseline hazard function can be modeled by means of a separate Cox regression term.

We investigated the marginal likelihood‐based estimation of the model parameters. This method works well for the Cox regression parameters, but underestimates the frailty variance and also may result in a biased estimate of the scale a of the baseline hazard function.

In general, the underestimation of the variance of the frailty in the survival models with random effect is a serious drawback of the maximum likelihood estimation. This problem was solved for the log‐normally‐distributed frailty through the use of the Laplace approximation technique for the marginal likelihood which treats both the fixed and the random effects to be estimated as the fixed ones. This approach provides three types of the estimates of the frailty variance: the best linear unbiased prediction (BLUP) estimate, the maximum likelihood (ML) estimate, and the restricted maximum likelihood (REML) estimate.^15^, ^26^, ^27^ In contrast to the first two estimates, the REML estimate can be unbiased.

Unfortunately, the REML estimate of the frailty variance $\sigma^{2}$ is not available in the case of the gamma‐distributed random effect.^16^ Further research is required to develop the methodology for unbiased estimation of the frailty variance for the distributions beyond the log‐normal.

In our simulations, it appears that the estimation biases of the frailty variance and of the scale parameter are proportional to the true value of $\sigma^{2}$ and inversely proportional to the number of clusters. The latter is due to a fact that the difference between ML and REML estimates is getting negligibly small when the number of clusters tends to infinity.

In real‐life applications that we studied so far,^9^, ^10^, ^11^ the frailty variance is usually rather low, and the number of clusters, such as the GP practices, is sufficiently high to make the marginal likelihood estimation of all the model parameters practically unbiased.

We also considered interval estimation and demonstrated that the profile likelihood‐based confidence intervals provide good coverage for all model parameters.

In the study of survival in T2DM, double‐Cox model provided an important insight into the slowing of the mortality improvement in the UK. This finding is corroborated by a recent study^28^ that found that the number of middle‐layer super output areas (MSOA) in England with a decline in life expectancy increased considerably in 2014‐2019 compared to 2010‐2014. In women, this decline was especially pronounced at 262%, compared to 11.5% decline in men.

The Associate Editor suggested that a comparatively high average age of over 61 years in our study participants may be due to a presence of left truncation. Overall, type 2 diabetes mellitus is more prevalent in the older ages compared to younger ages. In our study, T2DM diagnosis peaked between the ages 55 to 64 and 60 to 69 years for men and women, respectively. Across the world, in 2017, the diabetes prevalence peaked at 65‐69 years of age for men, while for women it peaked at the age of 70‐79 years. Prevalence varies by World Bank income group and geographical region, with 44% of cases in the over 65s in high‐income countries compared with 12% and 23% for low‐ and middle‐income countries.^29^ Importantly, almost half of all people (49.7%) living with diabetes were undiagnosed in 2017, counting for over 224 million adults (18–99 years). The percentage of undiagnosed T2DM varies from 37.6% in the North America and Caribbean region to 69.2% in the Africa region.^29^ The prolonged asymptomatic phase of type 2 diabetes may last many years.^30^ Therefore, onset date of T2DM is undoubtedly subject to left truncation. However, we believe that this does not affect our findings as the entry point to our study was not onset but diagnosis of T2DM.

Overall, the double‐Cox regression is a useful addition to the toolkit of survival analysis, and can be recommended for use in practice.

## Supporting information


**Appendix S1**: Supporting Information

## Data Availability

Our full simulation results are available as e‐print arXiv 2022; 2206.05141v1. stat.ME.^20^ An R package implementing the double‐Cox modeling of the survival data is available at Github, https://rdrr.io/github/AB5103/doubleCoxr/. The data used in the example on survival after a diagnosis of diabetes mellitus may be obtained from a third party and are not publicly available. For all interested researchers, THIN data are available via IQVIA, https://www.iqvia.com/, subject to ethical approval of the THIN Scientific Review Committee and governance controls.
