# Supplementary material for: A double‐Cox model for non‐proportional hazards survival analysis with frailty
Source: Stat Med. 2023 May 15;42(18):3114–27. doi: 10.1002/sim.9760 (PMC10946853; doi:10.1002/sim.9760)
Supplement: Supplementary file 1 — Appendix S1: Supporting Information [file SIM-42-3114-s001.pdf]

Supplementary materials

for

Alexander Begun, Elena Kulinskaya and Njabulo Ncube

A double-Cox model for non-proportional hazards survival analysis with frailty

A.1 Details of the EM and the PL algorithms

We denote a log-likelihood function corresponding to a particular likelihood function by using an additional symbol $\mathcal{L}$ in the log-likelihood function name (cf. $\mathcal{LL}_c$, $\mathcal{LL}_m$, etc.).

EM algorithm

The EM algorithm is an iterative procedure that involves four steps [?].

1. Estimate the conditional expectation of Z_i and ω_i given the observed data and the current vector of parameter estimates $\hat{\zeta}_\sigma$ by using Bayes' theorem;
2. Update the estimates of the Cox-regression parameters and parameters of the baseline hazard function $(a, b, \beta_{scale}, \beta_{shape})$ by maximizing the conditional log-likelihood $\mathcal{LL}_c(\text{Data}|a, b, \beta_{scale}, \beta_{shape}, \hat{\sigma}^2, \hat{Z}_1, ..., \hat{Z}_{N_{cl}})$ given observed data and the estimates of the frailties obtained in Step 1;
3. Update the estimate of σ^2 by maximizing $\sum_i \log f(\hat{Z}_i|\sigma^2)$;
4. Repeat (1)-(3) until convergence is reached.

Penalized likelihood

The penalized likelihood approach is an alternative to the EM-algorithm to obtain the ML estimates in the presence of the unobserved random effects. In the case of gamma-distributed frailties this approach results in the same parameter estimates as the EM-algorithm and involves iterative estimation of the random effects (log-frailties). The idea of the method is to exclude unlikely small or unlikely large values of the frailty

subtracting a penalty function $g(\omega_1, ..., \omega_{N_{cl}}|\sigma^2)$ from the conditional likelihood (in terms of the random effects $\omega_1, ..., \omega_{N_{cl}}$), i.e.

$$\mathcal{LL}_{pen}(\text{Data}|\zeta, \sigma^2, \omega_1, ..., \omega_{N_{cl}}) = \mathcal{LL}_c(\text{Data}|\zeta, \omega_1, ..., \omega_{N_{cl}}) - g(\omega_1, ..., \omega_{N_{cl}}|\sigma^2),$$

with $g(\omega_1, ..., \omega_{N_{cl}}|\sigma^2) = -(1/\sigma^2)\sum_{i=1}^{N_{cl}}(\omega_i - \exp(\omega_i))$. Define the log profile likelihood for σ^2 as the conditional log likelihood restricted to the one-dimensional function by substituting the vector-parameter $\hat{\zeta}(\sigma^2)$ for given σ^2.

The solution to the penalized log-likelihood coincides with the EM solution for any fixed value of σ^2 [?, ?]. Furthermore, the estimate of σ^2 can be found through maximizing the profile likelihood

$$\begin{aligned}\mathcal{LL}_{prof}(\text{Data}|\sigma^2) =& \mathcal{LL}_{pen}(\text{Data}|\hat{\zeta}, \sigma, \hat{\omega}_1, ..., \hat{\omega}_{N_{cl}}) \\ &+ \sum_{i=1}^{N_{cl}} \left(d_i + \gamma - (\gamma + d_i)\ln(\gamma + d_i) + \gamma\ln\gamma + \ln\frac{\Gamma(\gamma + d_i)}{\Gamma(\gamma)} \right),\end{aligned}$$

where $d_i = \sum_j \delta_{ij}$ and $\gamma = 1/\sigma^2$.

The algorithm for parameter estimation using the penalized likelihood approach includes three steps:

1. *Inner loop.* Update the estimate of the vector-parameter ζ and ω_i, $i = 1, ..., N_{cl}$, for current estimate of σ^2, maximazing the penalized log-likelihood $\mathcal{LL}_{pen}$;

2. *Outer loop.* Update the estimate of σ^2 for current estimates of the vector-parameter ζ and ω_i, $i = 1, ..., N_{cl}$, maximizing the profile log-likelihood $\mathcal{LL}_{prof}$;

3. Repeat (1)-(2) until the precision value for σ^2 is reached.

A.2 Designing a simulation with a predefined proportion of censoring

The time-to-failure $X = \min(T, C)$, where conditionally independent (given frailty and covariates) random variables T and C are survival and censoring times, respectively. We assume that the censoring time is uniformly distributed on an interval [0,θ] ($C \sim Uni(0, \theta)$) with probability density function $g(c|\theta) = \theta^{-1}$. For an individual i , P($T \geqslant$

$C|Z_i, \mathbf{u}_i)$ is the probability of being censored, and $\delta = Ind(C \leqslant T)$ is the censoring index. Then, (see [?])

$$\mathbb{P}(\delta = 1|\theta, Z_i, \mathbf{u}_i) = \mathbb{P}(C \leqslant T < \infty, 0 \leqslant C < \infty|Z_i, \mathbf{u}_i) = \int_0^\theta g(c|\theta) \int_c^\infty f(t|a, b, Z_i, \mathbf{u}_i) dtdc$$
$$= \theta^{-1} \int_0^\infty \int_c^\infty f(t|a, b, Z_i, \mathbf{u}_i) dtdc,$$

where $f(.)$ is the probability density function of the survival time. Applying this formula to the Weibull and the Gompertz double-Cox models, we obtain

$$\mathbb{P}(\delta = 1|\theta, Z_i, \mathbf{u}_i) = \frac{Z_i a e^{\beta_{scale}\mathbf{u}_i}}{\theta b e^{\beta_{shape}\mathbf{u}_i}} \int_0^{(\theta/Z_i a e^{\beta_{scale}\mathbf{u}_i})^{b e^{\beta_{shape}\mathbf{u}_i}}} x^{b^{-1} e^{-\beta_{shape}\mathbf{u}_i} - 1} e^{-x} dx$$
$$= \frac{Z_i a e^{\beta_{scale}\mathbf{u}_i}}{\theta b e^{\beta_{shape}\mathbf{u}_i}} \Gamma\left(b^{-1} e^{-\beta_{shape}\mathbf{u}_i}, (\theta/Z_i a e^{\beta_{scale}\mathbf{u}_i})^{b e^{\beta_{shape}\mathbf{u}_i}}\right),$$

in the case of the Weibull model and

$$\mathbb{P}(\delta = 1|\theta, Z_i, \mathbf{u}_i) = \frac{e^{Z_i a e^{\beta_{scale}\mathbf{u}_i}/b e^{\beta_{shape}\mathbf{u}_i}}}{\theta b e^{\beta_{shape}\mathbf{u}_i}}$$
$$\times \left(E_1(Z_i a e^{\beta_{scale}\mathbf{u}_i}/b e^{\beta_{shape}\mathbf{u}_i}) - E_1(Z_i a e^{\beta_{scale}\mathbf{u}_i} e^{b e^{\beta_{shape}\mathbf{u}_i}}/b e^{\beta_{shape}\mathbf{u}_i})\right),$$

where $E_1(x)$ is the exponential integral $E_1(x) = \int_x^\infty e^{-s} s^{-1} ds$, in the case of the Gompertz model.

The population censoring rate is obtained by averaging the individual censoring rates with respect to random variables Z and $\mathbf{u}$,

$$\mathbb{P}(\delta = 1|\theta) = \mathbb{E}_{Z,\mathbf{u}} \mathbb{P}(\delta = 1|\theta, Z, \mathbf{u}) = \int_{D_u \times D_z} P(\delta = 1|\theta, Z, \mathbf{u}) p_Z(z) p_U(u) dz du,$$

where $p_U(u)$ and $p_Z(z)$ is the probability density functions for independent random variables $\mathbf{U}$ and Z defined in the domains D_u and D_z, respectively. There is no simple closed form for the last integral. We use the Monte-Carlo integration

$$P(\delta = 1|\theta) \approx N_{sim}^{-1} \sum_{i=1}^{N_{sim}} P(\delta = 1|\theta, Z_i, \mathbf{u}_i) \quad \text{(A.1)}$$

for independent random pairs $(Z_i, \mathbf{u}_i)$. This approximation converges to $P(\delta = 1|\theta)$ as $N_{sim} \to \infty$ in accordance with the law of large numbers.

Given the population censoring rates p_{cens}, the unknown parameter θ is a numerical solution to the equation

$$\mathbb{P}(\delta = 1|\theta) = p_{cens}. \quad \text{(A.2)}$$

In simulations, the censoring times c_i for individuals $i = 1, \cdots, N$, are generated from $Uni(0, \theta)$ distribution. For the survival times t_i, define $s_i \sim U(0, 1)$. The survival time t_i for an individual i is generated as

$$t_i = a \left(-\frac{\log(s_i)}{Z_i e^{\beta_{scale}\mathbf{u}_i}} \right)^{1/be^{\beta_{shape}\mathbf{u}_i}}$$

in the case of the Weibull model and as

$$t_i = \frac{1}{be^{\beta_{shape}\mathbf{u}_i}} \log \left(-\frac{be^{\beta_{shape}\mathbf{u}_i} \log(s_i)}{ae^{\beta_{scale}\mathbf{u}_i} Z_i} + 1 \right)$$

in the case of the Gompertz model.

A.3 Additional tables

N	σ^2	N_{cl}	%cens	$\beta_{success-scale}$		$\beta_{success-shape}$	$\beta_{score-scale}$		$\beta_{score-shape}$	σ^2	
				single Cox (parfm)	double Cox	double Cox	single Cox (parfm)	double Cox	double Cox	single Cox (parfm)	double Cox
300	0	10	0	$-5.59\cdot10^{-3}$($-5.58\cdot10^{-3}$)	$-1.00\cdot10^{-2}$	$1.47\cdot10^{-3}$	$-1.04\cdot10^{-2}$($-1.04\cdot10^{-2}$)	$-1.54\cdot10^{-2}$	$6.62\cdot10^{-3}$	$6.96\cdot10^{-6}$($4.99\cdot10^{-7}$)	$7.79\cdot10^{-6}$
			40	$-7.27\cdot10^{-3}$($-7.26\cdot10^{-3}$)	$-9.43\cdot10^{-3}$	$-1.22\cdot10^{-3}$	$-1.40\cdot10^{-2}$($-1.40\cdot10^{-2}$)	$-1.55\cdot10^{-2}$	$-1.10\cdot10^{-2}$	$1.02\cdot10^{-5}$($6.64\cdot10^{-7}$)	$1.15\cdot10^{-5}$
		100	0	$-9.43\cdot10^{-3}$($-9.42\cdot10^{-3}$)	$-1.21\cdot10^{-2}$	$-1.21\cdot10^{-3}$	$-1.88\cdot10^{-2}$($-1.88\cdot10^{-2}$)	$-2.17\cdot10^{-2}$	$5.60\cdot10^{-3}$	$6.50\cdot10^{-5}$($6.75\cdot10^{-6}$)	$6.79\cdot10^{-5}$
			40	$-1.32\cdot10^{-2}$($-1.32\cdot10^{-2}$)	$-1.53\cdot10^{-2}$	$-3.28\cdot10^{-3}$	$-3.74\cdot10^{-2}$($-3.74\cdot10^{-2}$)	$-3.96\cdot10^{-2}$	$-9.76\cdot10^{-3}$	$1.12\cdot10^{-4}$($1.13\cdot10^{-5}$)	$1.32\cdot10^{-4}$
	2	10	0	$-3.86\cdot10^{-3}$($-3.86\cdot10^{-3}$)	$-8.53\cdot10^{-3}$	$2.39\cdot10^{-4}$	$-4.93\cdot10^{-3}$($-4.93\cdot10^{-3}$)	$-7.32\cdot10^{-3}$	$9.92\cdot10^{-5}$	$-3.03\cdot10^{-1}$($-3.03\cdot10^{-1}$)	$-2.96\cdot10^{-1}$
			40	$-4.29\cdot10^{-3}$($-4.29\cdot10^{-3}$)	$-8.37\cdot10^{-3}$	$1.94\cdot10^{-4}$	$-1.17\cdot10^{-2}$($-1.17\cdot10^{-2}$)	$-1.74\cdot10^{-2}$	$-2.15\cdot10^{-3}$	$-2.70\cdot10^{-1}$($-2.70\cdot10^{-1}$)	$-2.60\cdot10^{-1}$
		100	0	$-3.78\cdot10^{-3}$($-3.78\cdot10^{-3}$)	$-8.76\cdot10^{-3}$	$4.31\cdot10^{-4}$	$-1.41\cdot10^{-2}$($-1.41\cdot10^{-2}$)	$-2.21\cdot10^{-2}$	$3.80\cdot10^{-3}$	$-1.81\cdot10^{-2}$($-1.81\cdot10^{-2}$)	$-8.56\cdot10^{-3}$
			40	$-7.19\cdot10^{-3}$($-7.19\cdot10^{-3}$)	$-1.02\cdot10^{-2}$	$-2.75\cdot10^{-3}$	$5.04\cdot10^{-3}$($5.04\cdot10^{-3}$)	$-1.73\cdot10^{-4}$	$3.16\cdot10^{-3}$	$-4.05\cdot10^{-3}$($-4.06\cdot10^{-3}$)	$1.06\cdot10^{-2}$
1000	0	10	0	$-1.91\cdot10^{-3}$($-1.90\cdot10^{-3}$)	$-2.89\cdot10^{-3}$	$-2.63\cdot10^{-5}$	$-5.68\cdot10^{-3}$($-5.68\cdot10^{-3}$)	$-6.86\cdot10^{-3}$	$2.08\cdot10^{-3}$	$3.17\cdot10^{-6}$($3.40\cdot10^{-7}$)	$3.68\cdot10^{-6}$
			40	$-1.98\cdot10^{-3}$($-1.97\cdot10^{-3}$)	$-2.51\cdot10^{-3}$	$-1.14\cdot10^{-3}$	$-1.07\cdot10^{-2}$($-1.07\cdot10^{-2}$)	$-1.12\cdot10^{-2}$	$-5.27\cdot10^{-3}$	$4.39\cdot10^{-6}$($3.96\cdot10^{-7}$)	$5.02\cdot10^{-6}$
		100	0	$-9.62\cdot10^{-4}$($-9.57\cdot10^{-4}$)	$-2.16\cdot10^{-3}$	$2.52\cdot10^{-4}$	$-5.91\cdot10^{-3}$($-5.90\cdot10^{-3}$)	$-6.35\cdot10^{-3}$	$9.61\cdot10^{-4}$	$3.07\cdot10^{-5}$($4.74\cdot10^{-6}$)	$3.32\cdot10^{-5}$
			40	$-4.97\cdot10^{-3}$($-4.97\cdot10^{-3}$)	$-5.47\cdot10^{-3}$	$-1.13\cdot10^{-3}$	$-1.17\cdot10^{-2}$($-1.17\cdot10^{-2}$)	$-1.18\cdot10^{-2}$	$-7.37\cdot10^{-3}$	$5.42\cdot10^{-5}$($7.86\cdot10^{-6}$)	$6.21\cdot10^{-5}$
	2	10	0	$-3.19\cdot10^{-3}$($-3.19\cdot10^{-3}$)	$-3.29\cdot10^{-3}$	$-5.07\cdot10^{-4}$	$-4.49\cdot10^{-3}$($-4.49\cdot10^{-3}$)	$-3.39\cdot10^{-3}$	$-7.66\cdot10^{-4}$	$-2.99\cdot10^{-1}$($-2.99\cdot10^{-1}$)	$-2.97\cdot10^{-1}$
			40	$-1.82\cdot10^{-3}$($-1.82\cdot10^{-3}$)	$-3.32\cdot10^{-3}$	$1.17\cdot10^{-4}$	$-1.69\cdot10^{-3}$($-1.69\cdot10^{-3}$)	$-1.38\cdot10^{-3}$	$-1.52\cdot10^{-3}$	$-2.92\cdot10^{-1}$($-2.92\cdot10^{-1}$)	$-2.89\cdot10^{-1}$
		100	0	$-2.47\cdot10^{-3}$($-2.47\cdot10^{-3}$)	$-2.53\cdot10^{-3}$	$-5.65\cdot10^{-4}$	$-1.27\cdot10^{-3}$($-1.27\cdot10^{-3}$)	$-2.38\cdot10^{-3}$	$2.28\cdot10^{-4}$	$-3.16\cdot10^{-2}$($-3.16\cdot10^{-2}$)	$-2.90\cdot10^{-2}$
			40	$5.17\cdot10^{-4}$($5.17\cdot10^{-4}$)	$-5.39\cdot10^{-4}$	$-1.79\cdot10^{-4}$	$-6.00\cdot10^{-3}$($-6.00\cdot10^{-3}$)	$-7.18\cdot10^{-3}$	$7.18\cdot10^{-4}$	$-2.05\cdot10^{-2}$($-2.05\cdot10^{-2}$)	$-1.69\cdot10^{-2}$

Table A.1: Bias in the over-parametrised Weibull model by sample size n, σ^2, the number of clusters N_{cl}, and the percent of censored subjects. True values of the parameters: $\beta_{scale-Success} = -0.5$, $\beta_{scale-score} = -1$, $\beta_{shape-Success} = 0$, $\beta_{shape-score} = 0$, $p_{Success} = 0.5$.

N	σ^2	N_{cl}	%cens	$\beta_{success-scale}$		$\beta_{success-shape}$	$\beta_{score-scale}$		$\beta_{score-shape}$	σ^2	
				single Cox (parfm)	double Cox	double Cox	single Cox (parfm)	double Cox	double Cox	single Cox (parfm)	double Cox
300	0	10	0	$-3.60\cdot10^{-3}(-3.60\cdot10^{-3})$	$2.72\cdot10^{-3}$	$-1.30\cdot10^{-3}$	$-9.49\cdot10^{-3}(-9.49\cdot10^{-3})$	$2.42\cdot10^{-2}$	$-5.48\cdot10^{-3}$	$7.41\cdot10^{-6}(4.45\cdot10^{-7})$	$8.09\cdot10^{-6}$
			40	$-1.28\cdot10^{-2}(-1.27\cdot10^{-2})$	$2.29\cdot10^{-2}$	$-5.19\cdot10^{-3}$	$-1.94\cdot10^{-2}(-1.93\cdot10^{-2})$	$5.14\cdot10^{-3}$	$-4.69\cdot10^{-3}$	$1.03\cdot10^{-5}(7.32\cdot10^{-7})$	$1.16\cdot10^{-5}$
		100	0	$-7.03\cdot10^{-3}(-7.01\cdot10^{-3})$	$-2.81\cdot10^{-4}$	$-9.85\cdot10^{-4}$	$-1.50\cdot10^{-2}(-1.50\cdot10^{-2})$	$4.47\cdot10^{-2}$	$-8.93\cdot10^{-3}$	$7.35\cdot10^{-5}(6.55\cdot10^{-6})$	$7.47\cdot10^{-5}$
			40	$-1.29\cdot10^{-2}(-1.29\cdot10^{-2})$	$-4.12\cdot10^{-3}$	$-1.66\cdot10^{-3}$	$-2.04\cdot10^{-2}(-2.04\cdot10^{-2})$	$2.68\cdot10^{-2}$	$-7.55\cdot10^{-3}$	$8.75\cdot10^{-5}(8.37\cdot10^{-6})$	$7.75\cdot10^{-5}$
	2	10	0	$-2.54\cdot10^{-3}(-2.54\cdot10^{-3})$	$-8.80\cdot10^{-3}$	$3.75\cdot10^{-4}$	$-7.52\cdot10^{-3}(-7.52\cdot10^{-3})$	$-5.63\cdot10^{-2}$	$5.43\cdot10^{-3}$	$-3.04\cdot10^{-1}(-3.04\cdot10^{-1})$	$-2.96\cdot10^{-1}$
			40	$-6.16\cdot10^{-3}(-6.16\cdot10^{-3})$	$-1.92\cdot10^{-2}$	$1.36\cdot10^{-3}$	$-2.31\cdot10^{-2}(-2.31\cdot10^{-2})$	$-2.75\cdot10^{-2}$	$-1.64\cdot10^{-4}$	$-2.98\cdot10^{-1}(-2.98\cdot10^{-1})$	$-2.88\cdot10^{-1}$
		100	0	$-7.74\cdot10^{-3}(-7.74\cdot10^{-3})$	$-1.43\cdot10^{-2}$	$3.60\cdot10^{-4}$	$-1.29\cdot10^{-2}(-1.29\cdot10^{-2})$	$-4.20\cdot10^{-2}$	$2.80\cdot10^{-3}$	$-1.77\cdot10^{-2}(-1.77\cdot10^{-2})$	$-8.75\cdot10^{-3}$
			40	$-9.15\cdot10^{-3}(-9.15\cdot10^{-3})$	$-1.33\cdot10^{-2}$	$1.32\cdot10^{-4}$	$-9.14\cdot10^{-3}(-9.14\cdot10^{-3})$	$-7.04\cdot10^{-3}$	$-1.61\cdot10^{-3}$	$-1.50\cdot10^{-2}(-1.50\cdot10^{-2})$	$-2.37\cdot10^{-3}$
1000	0	10	0	$2.31\cdot10^{-5}(2.72\cdot10^{-5})$	$-2.11\cdot10^{-3}$	$8.34\cdot10^{-5}$	$-4.38\cdot10^{-3}(-4.38\cdot10^{-3})$	$-2.52\cdot10^{-3}$	$-4.57\cdot10^{-4}$	$3.56\cdot10^{-6}(1.98\cdot10^{-7})$	$4.00\cdot10^{-6}$
			40	$-2.43\cdot10^{-3}(-2.42\cdot10^{-3})$	$-4.62\cdot10^{-3}$	$2.26\cdot10^{-4}$	$-4.92\cdot10^{-3}(-4.92\cdot10^{-3})$	$2.47\cdot10^{-3}$	$-1.20\cdot10^{-3}$	$4.64\cdot10^{-6}(2.58\cdot10^{-7})$	$5.32\cdot10^{-6}$
		100	0	$-2.49\cdot10^{-3}(-2.48\cdot10^{-3})$	$-7.33\cdot10^{-3}$	$6.56\cdot10^{-4}$	$-6.02\cdot10^{-3}(-6.02\cdot10^{-3})$	$-9.13\cdot10^{-4}$	$-6.66\cdot10^{-4}$	$3.64\cdot10^{-5}(3.21\cdot10^{-6})$	$3.73\cdot10^{-5}$
			40	$-4.67\cdot10^{-3}(-4.65\cdot10^{-3})$	$-4.63\cdot10^{-4}$	$-7.43\cdot10^{-4}$	$-1.03\cdot10^{-2}(-1.03\cdot10^{-2})$	$2.67\cdot10^{-2}$	$-5.78\cdot10^{-3}$	$4.38\cdot10^{-5}(3.90\cdot10^{-6})$	$4.63\cdot10^{-5}$
	2	10	0	$-2.22\cdot10^{-3}(-2.22\cdot10^{-3})$	$-8.25\cdot10^{-4}$	$-2.26\cdot10^{-4}$	$-4.19\cdot10^{-3}(-4.19\cdot10^{-3})$	$1.67\cdot10^{-4}$	$-6.40\cdot10^{-4}$	$-2.98\cdot10^{-1}(-2.98\cdot10^{-1})$	$-2.96\cdot10^{-1}$
			40	$-1.11\cdot10^{-3}(-1.11\cdot10^{-3})$	$-2.90\cdot10^{-3}$	$1.03\cdot10^{-4}$	$2.21\cdot10^{-3}(2.21\cdot10^{-3})$	$5.32\cdot10^{-4}$	$-8.38\cdot10^{-5}$	$-3.11\cdot10^{-1}(-3.11\cdot10^{-1})$	$-3.08\cdot10^{-1}$
		100	0	$-1.73\cdot10^{-3}(-1.73\cdot10^{-3})$	$-6.96\cdot10^{-3}$	$5.97\cdot10^{-4}$	$1.60\cdot10^{-3}(1.60\cdot10^{-3})$	$-3.28\cdot10^{-4}$	$8.33\cdot10^{-5}$	$-3.20\cdot10^{-2}(-3.20\cdot10^{-2})$	$-2.94\cdot10^{-2}$
			40	$-2.17\cdot10^{-4}(-2.17\cdot10^{-4})$	$-5.29\cdot10^{-3}$	$5.64\cdot10^{-4}$	$-3.65\cdot10^{-3}(-3.65\cdot10^{-3})$	$9.01\cdot10^{-3}$	$-2.00\cdot10^{-3}$	$-2.33\cdot10^{-2}(-2.33\cdot10^{-2})$	$-2.00\cdot10^{-2}$

Table A.2: Bias in the over-parametrised Gompertz model by sample size n, σ^2, the number of clusters N_{cl}, and the percent of censored subjects. True values: $\beta_{scale-Success} = -0.5$, $\beta_{scale-score} = -1$, $\beta_{shape-Success} = 0$, $\beta_{shape-score} = 0$, $p_{Success} = 0.5$.

A.4 Additional figures

A.4.1 Biases in parameter estimation

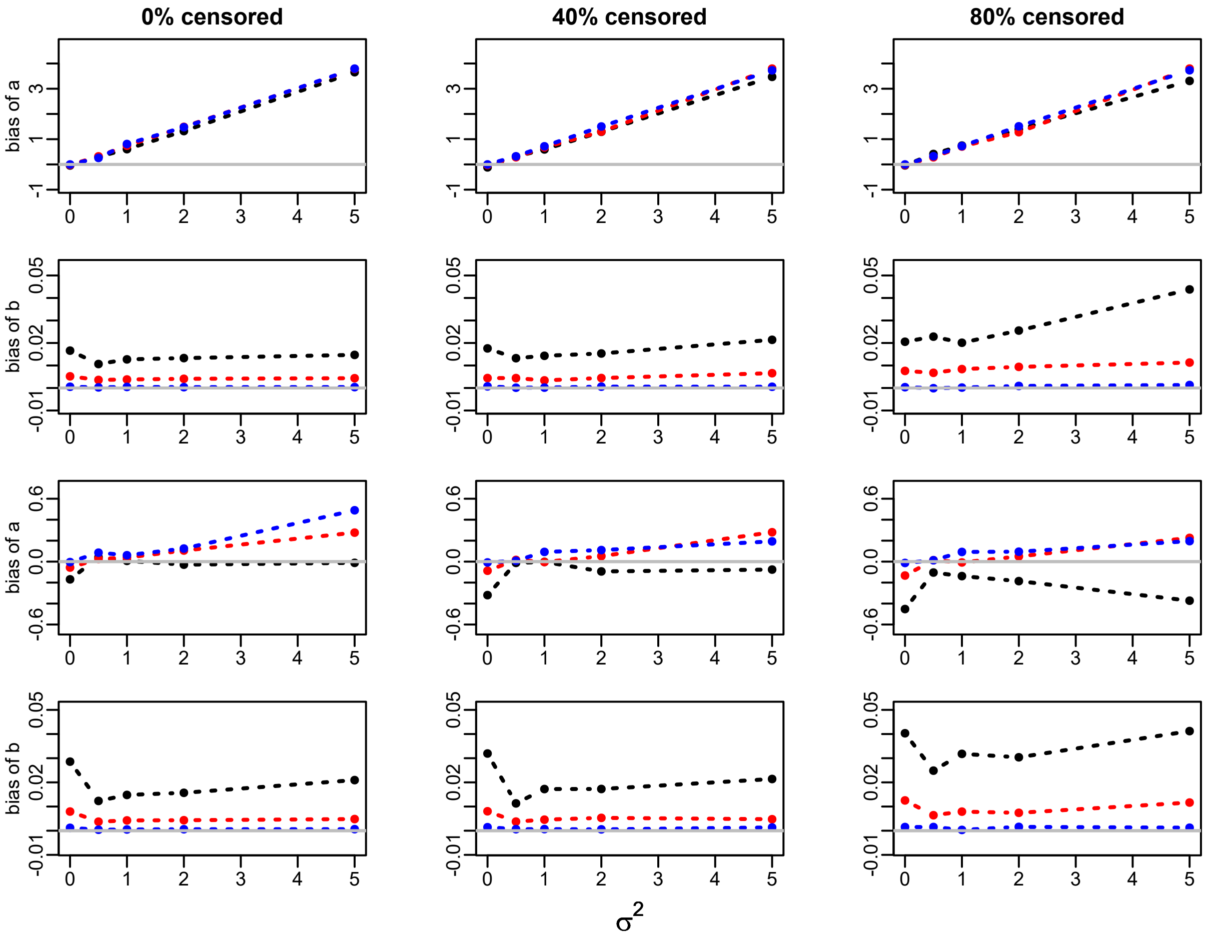

Top two rows: 10 clusters; bottom two rows: 100 clusters.

Figure A.1: Bias of the estimation of a and b parameters in the Weibull model. Success proportion= 0.25. Sample sizes: 300 (black), 1000 (red) and 10000 (blue). True values: $\beta_{success-scale}$ = -0.5 , $\beta_{success-shape}$= 0.05 , $\beta_{score-scale}$= -1 , $\beta_{score-shape}$= 0.1.

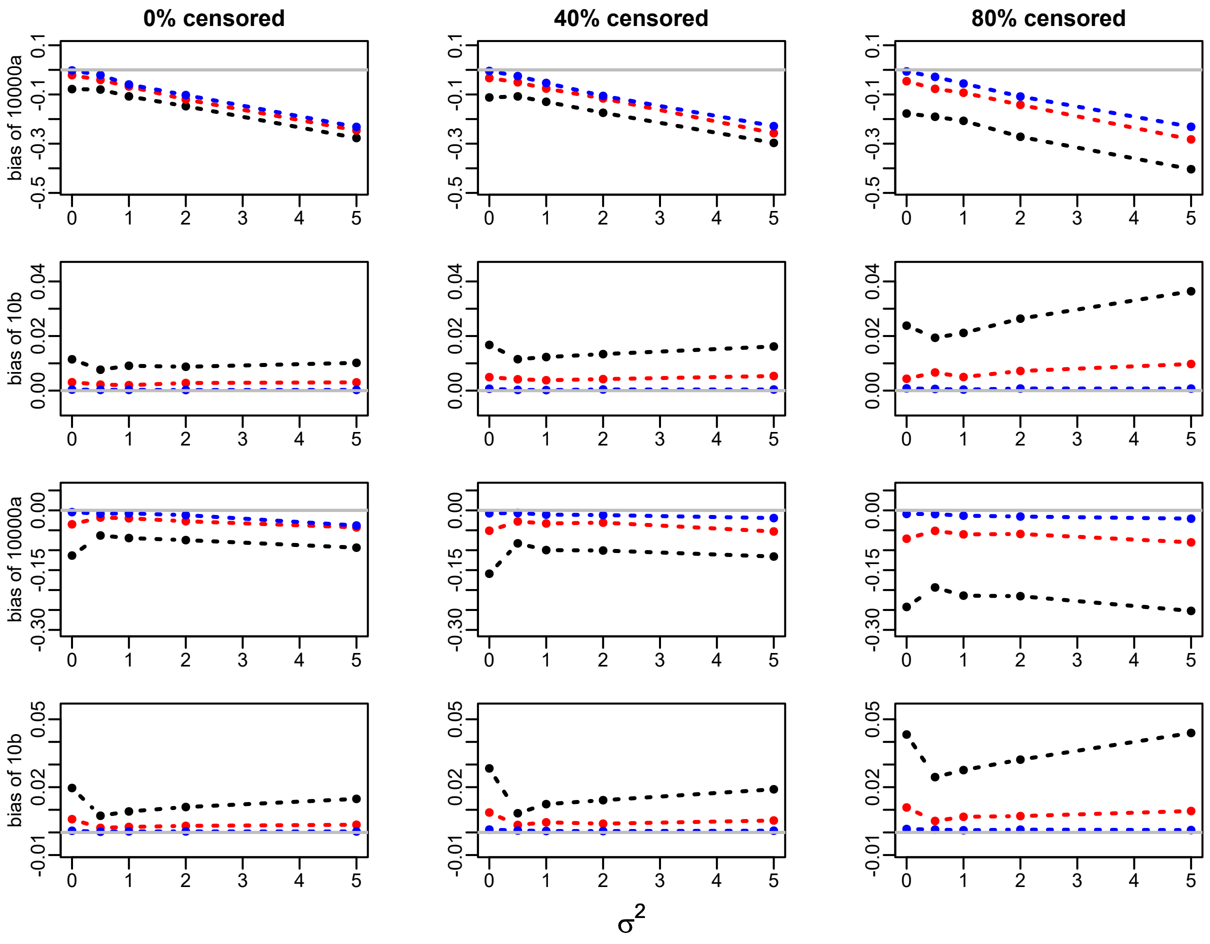

Top two rows: 10 clusters; bottom two rows: 100 clusters.

Figure A.2: Bias of the estimation of a and b parameters in the Gompertz model. Success proportion= 0.25 . 10 clusters. Sample sizes: 300 (black), 1000 (red) and 10000 (blue). True values: $\beta_{success-scale} = 0.5$, $\beta_{success-shape}$= 0.05 , $\beta_{score-scale}$= 1 , $\beta_{score-shape}$= 0.1.

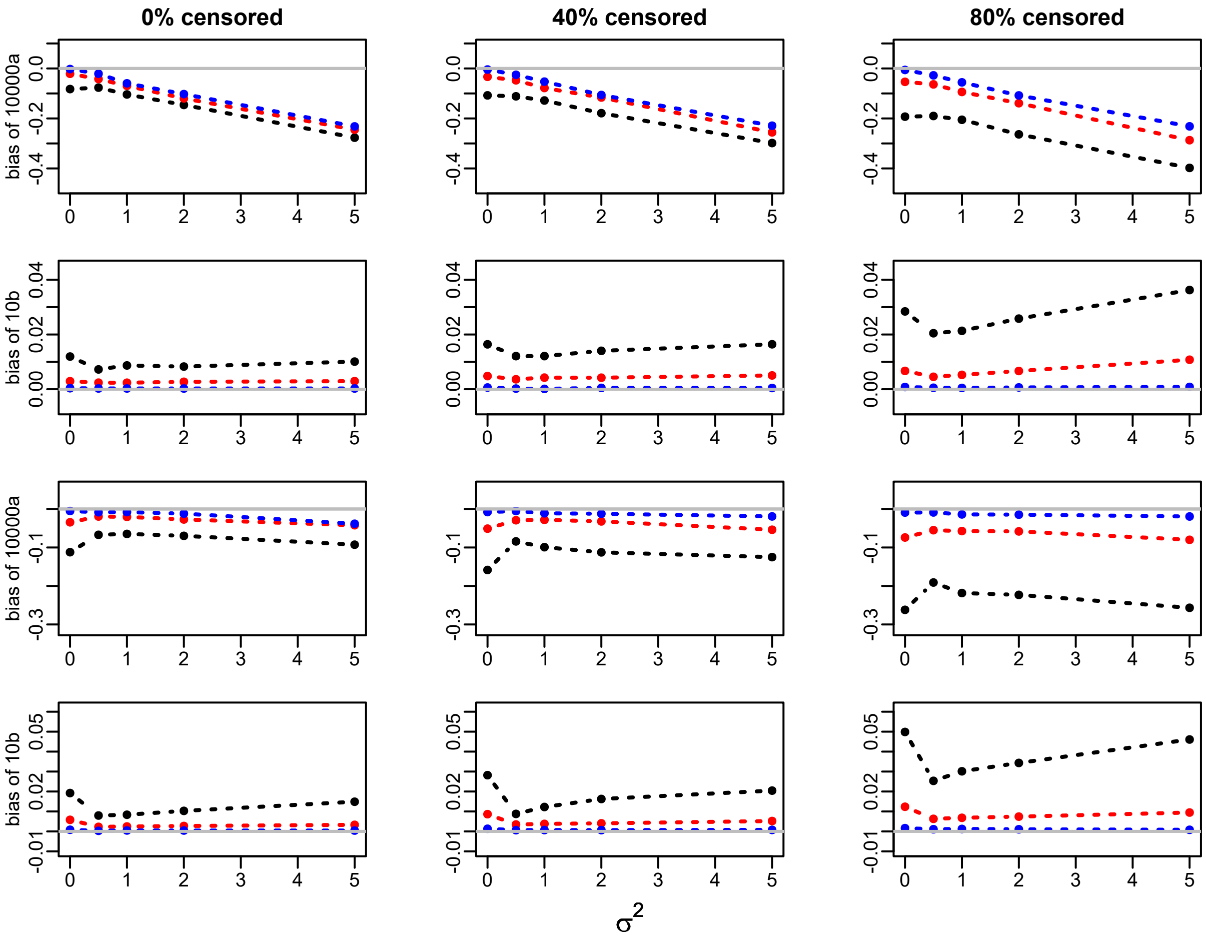

Figure A.3: Bias of the estimation of a and b parameters in the Gompertz model. Success proportion= 0.25 . 10 clusters. Sample sizes: 300 (black), 1000 (red) and 10000 (blue). True values: $\beta_{success-scale}$ = -0.5, $\beta_{success-shape}$= 0.05 , $\beta_{score-scale}$= -1 , $\beta_{score-shape}$= 0.1. Top two rows: 10 clusters; bottom two rows: 100 clusters.

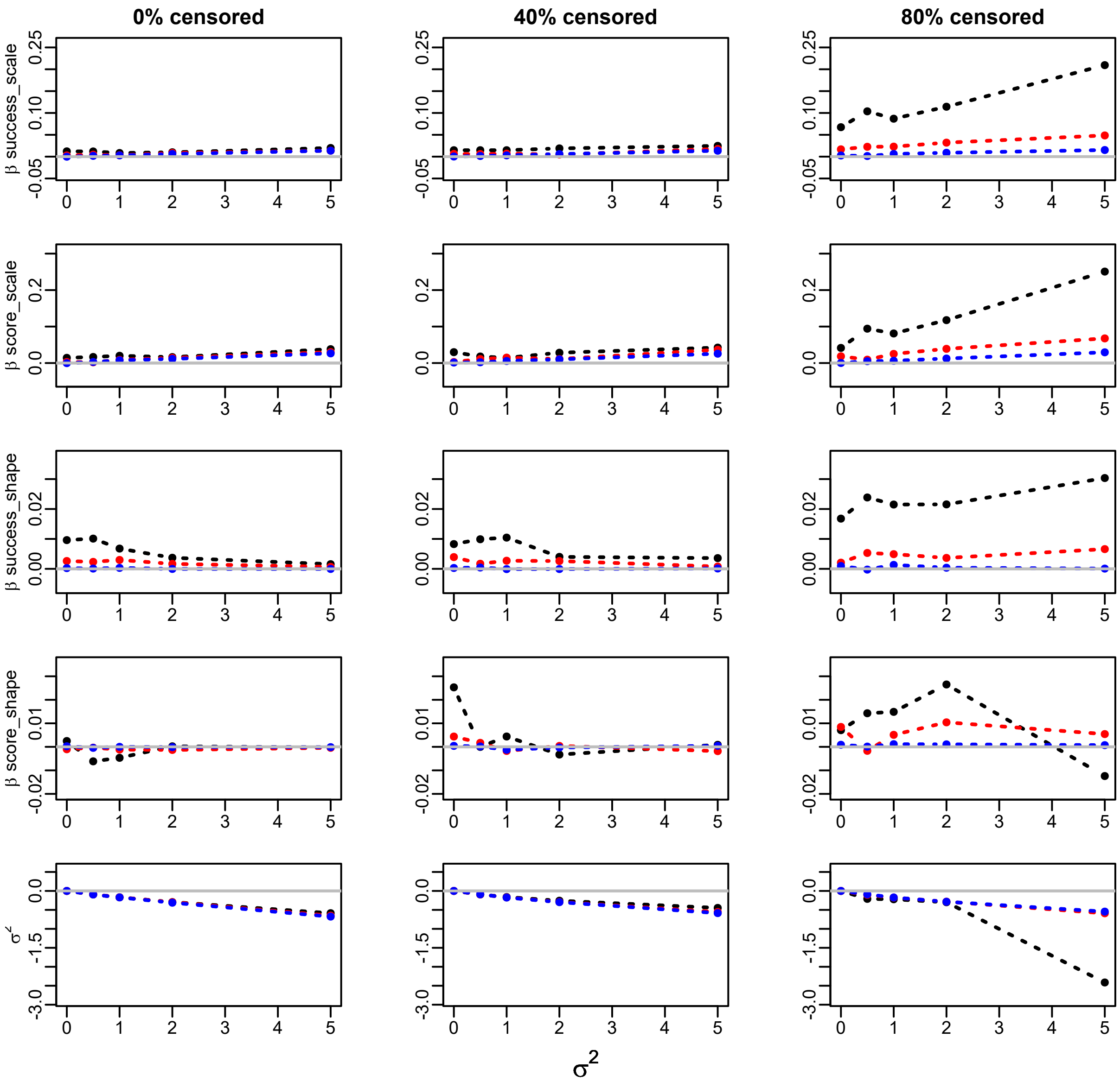

Figure A.4: Bias of the estimation of the Cox regression parameters and σ^2 in the Weibull model. Success proportion= 0.25. 10 clusters. Sample sizes: 300 (black), 1000 (red) and 10000 (blue). True values: $\beta_{success-scale}$ = 0.5 , $\beta_{success-shape}$= 0.05 , $\beta_{score-scale}$= 1 , $\beta_{score-shape}$= 0.1

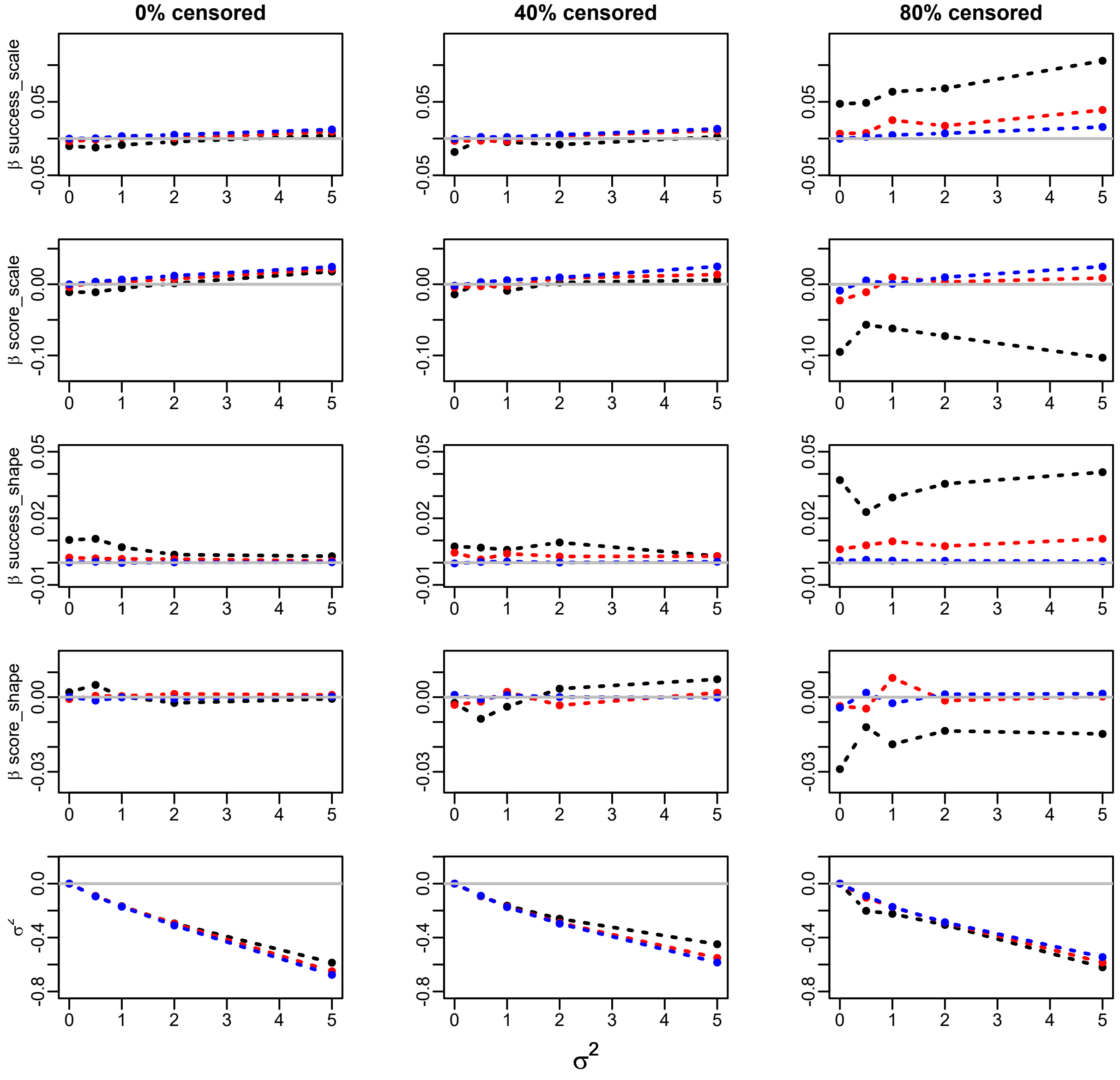

Figure A.5: Bias of the estimation of the Cox regression parameters and σ^2 in the Weibull model. Success proportion= 0.25. 10 clusters. Sample sizes: 300 (black), 1000 (red) and 10000 (blue). True values: $\beta_{success-scale}$ = -0.5 , $\beta_{success-shape}$= 0.05 , $\beta_{score-scale}$= -1 , $\beta_{score-shape}$= 0.1

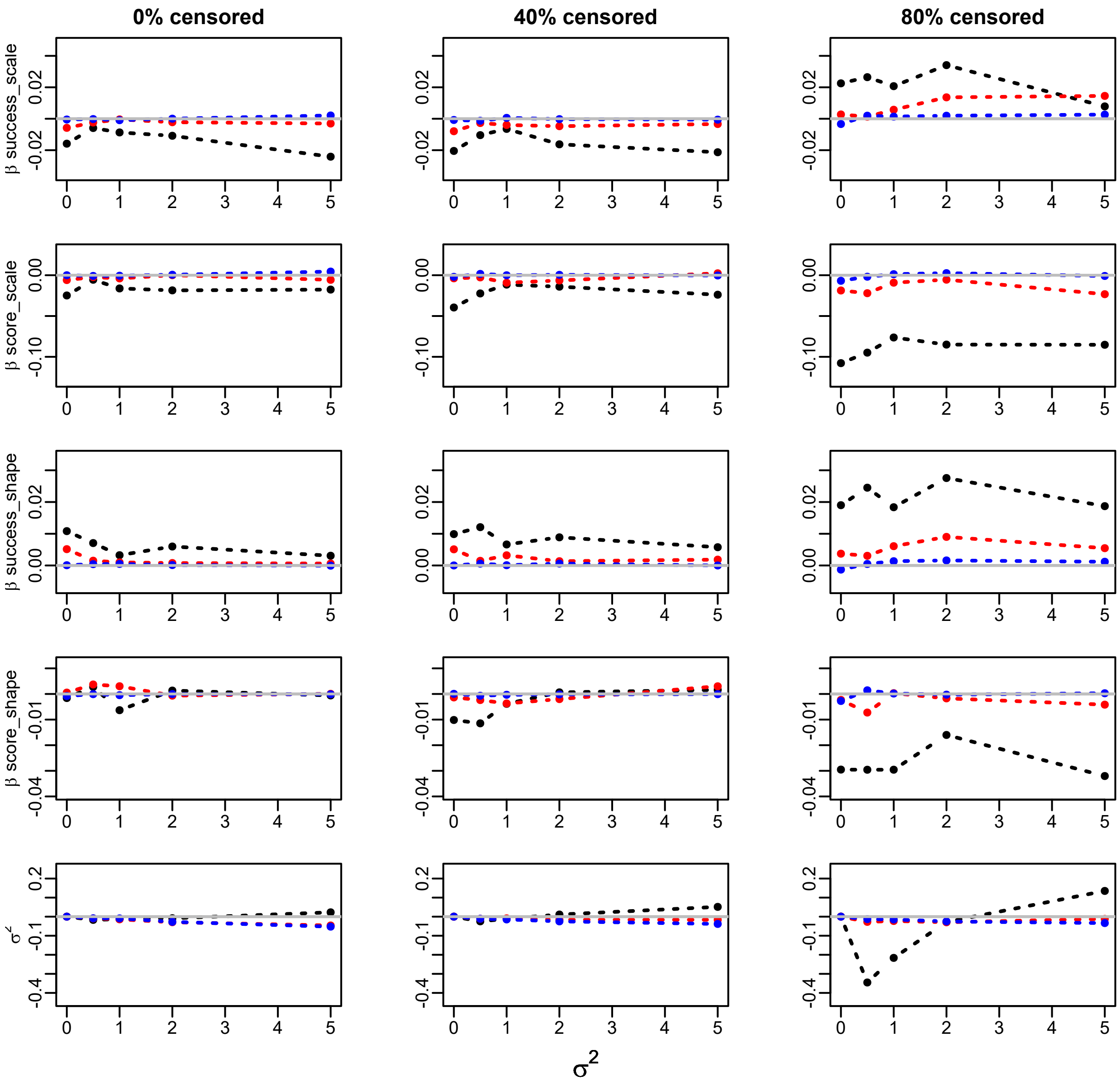

Figure A.6: Bias of the estimation of the Cox regression parameters and σ^2 in the Weibull model. Success proportion= 0.25. 100 clusters. Sample sizes: 300 (black), 1000 (red) and 10000 (blue). True values: $\beta_{success-scale}$ =- 0.5, $\beta_{success-shape}$= 0.05 , $\beta_{score-scale}$= -1 , $\beta_{score-shape}$= 0.1

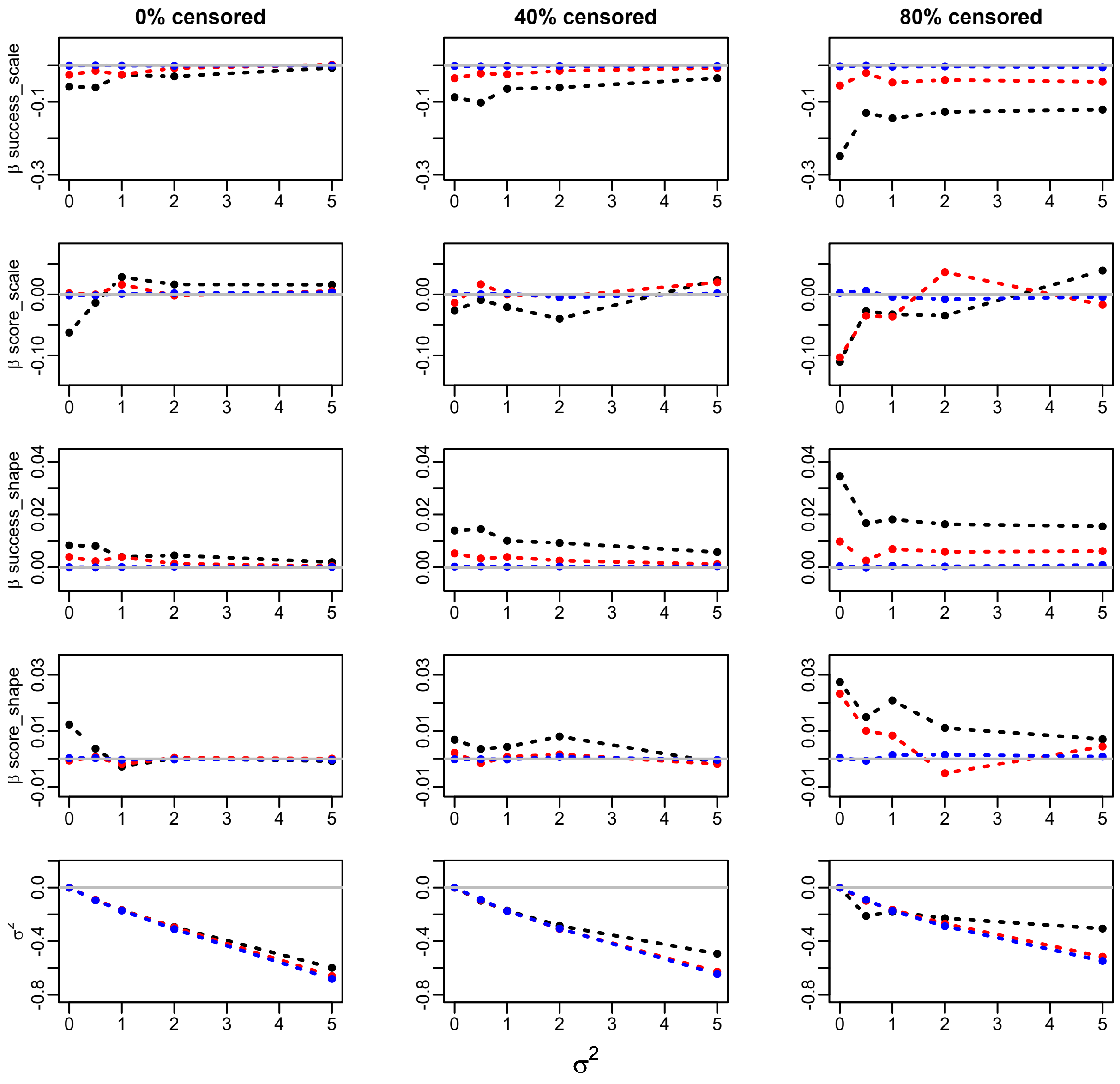

Figure A.7: Bias of the estimation of the Cox regression parameters and σ^2 in the Gompertz model. Success proportion= 0.25. 10 clusters. Sample sizes: 300 (black), 1000 (red) and 10000 (blue). True values: $\beta_{success-scale} = 0.5$, $\beta_{success-shape}= 0.05$, $\beta_{score-scale}= 1$, $\beta_{score-shape}= 0.1$

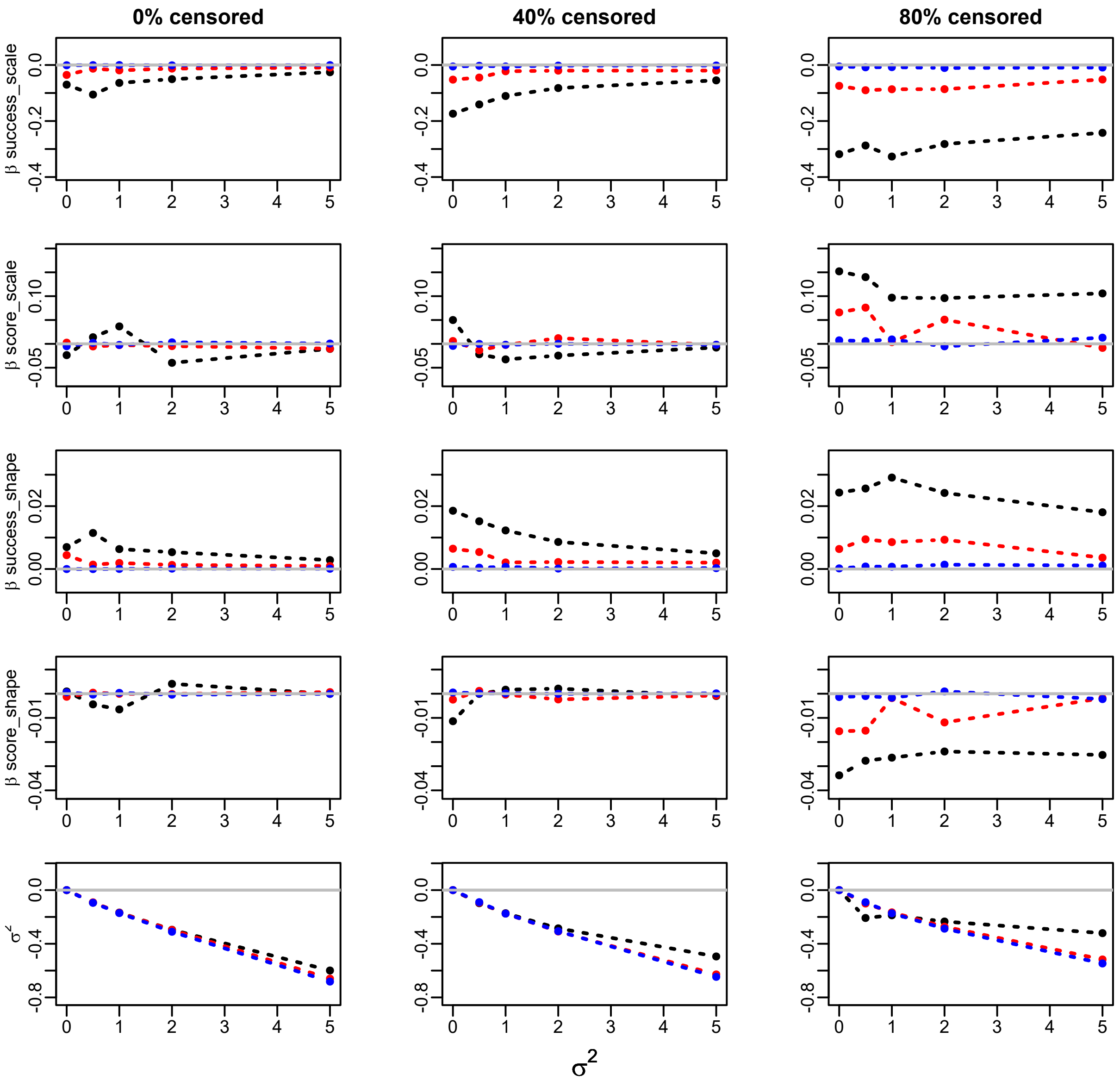

Figure A.8: Bias of the estimation of the Cox regression parameters and σ^2 in the Gompertz model. Success proportion= 0.25. 10 clusters. Sample sizes: 300 (black), 1000 (red) and 10000 (blue). True values: $\beta_{success-scale}$ = -0.5, $\beta_{success-shape}$= +0.05 , $\beta_{score-scale}$= -1 , $\beta_{score-shape}$=+ 0.1

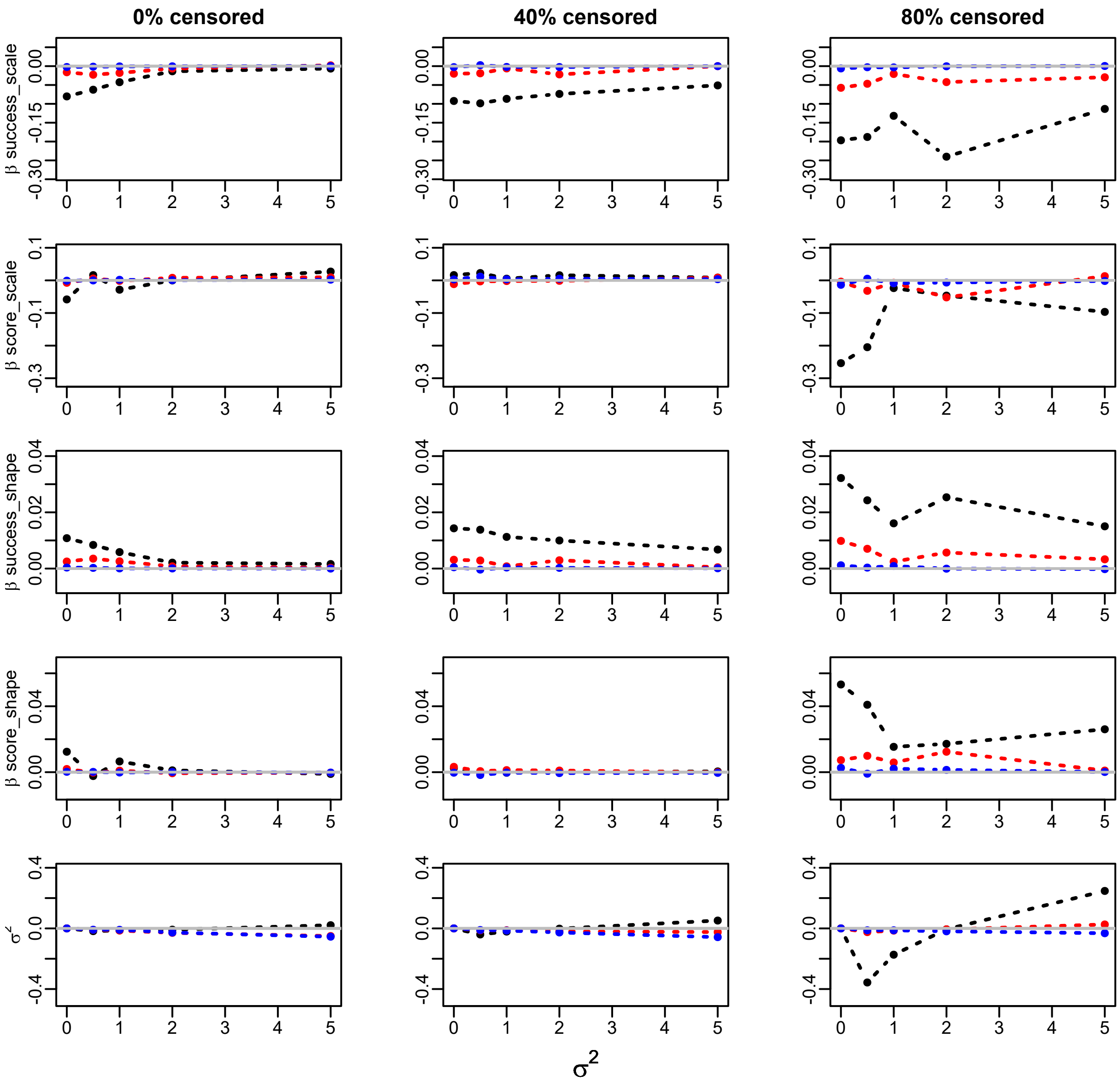

Figure A.9: Bias of the estimation of the Cox regression parameters and σ^2 in the Gompertz model. Success proportion= 0.25. 100 clusters. Sample sizes: 300 (black), 1000 (red) and 10000 (blue). True values: $\beta_{success-scale} = 0.5$, $\beta_{success-shape}= 0.05$, $\beta_{score-scale}= 1$, $\beta_{score-shape}= 0.1$

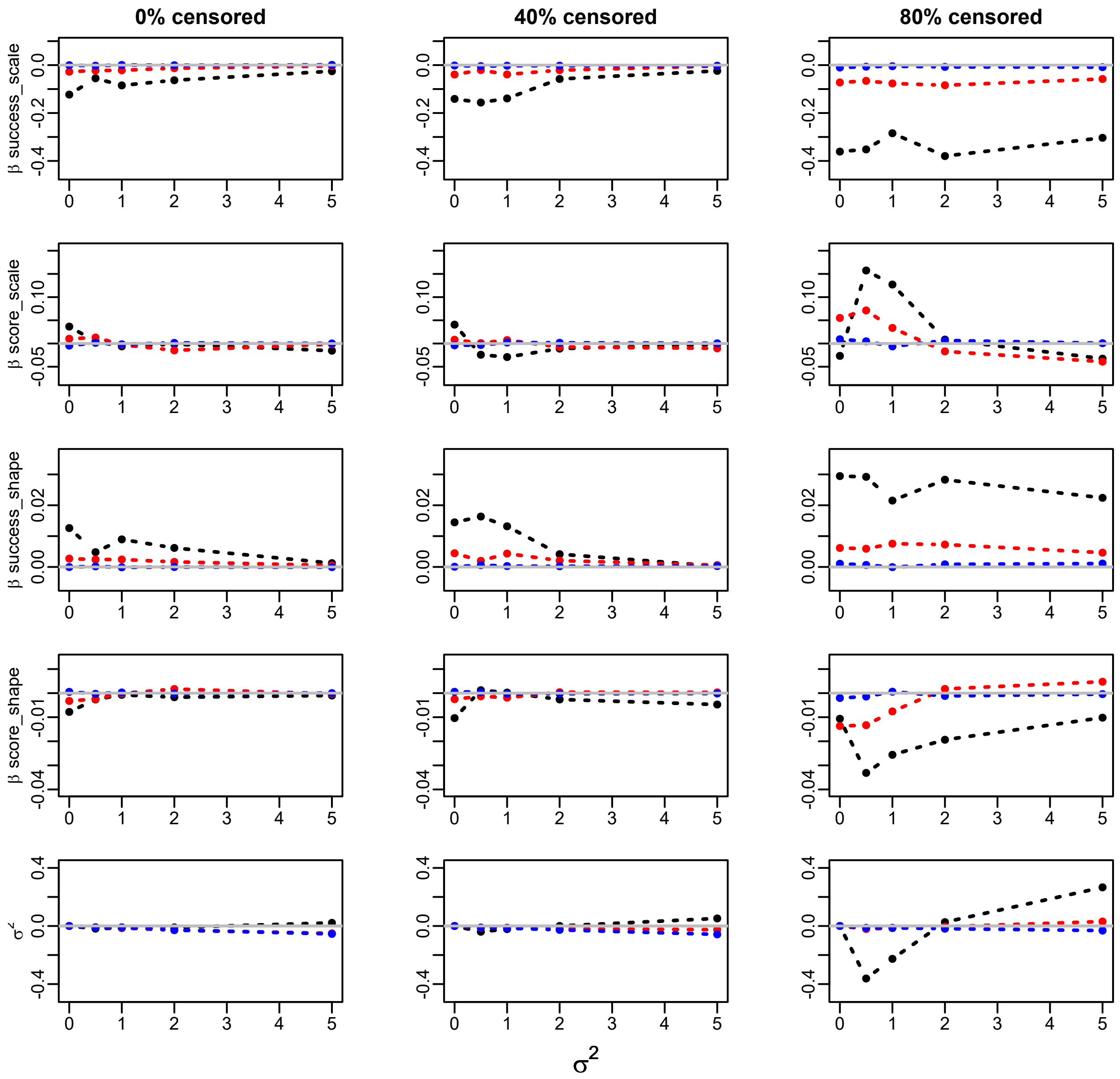

Figure A.10: Bias of the estimation of the Cox regression parameters and σ^2 in the Gompertz model. Success proportion= 0.25. 100 clusters. Sample sizes: 300 (black), 1000 (red) and 10000 (blue). True values: $\beta_{success-scale}$ = -0.5, $\beta_{success-shape}$= 0.05 , $\beta_{score-scale}$= -1 , $\beta_{score-shape}$= 0.1

A.4.2 Coverage of the SE and the PL confidence intervals for the Weibull model

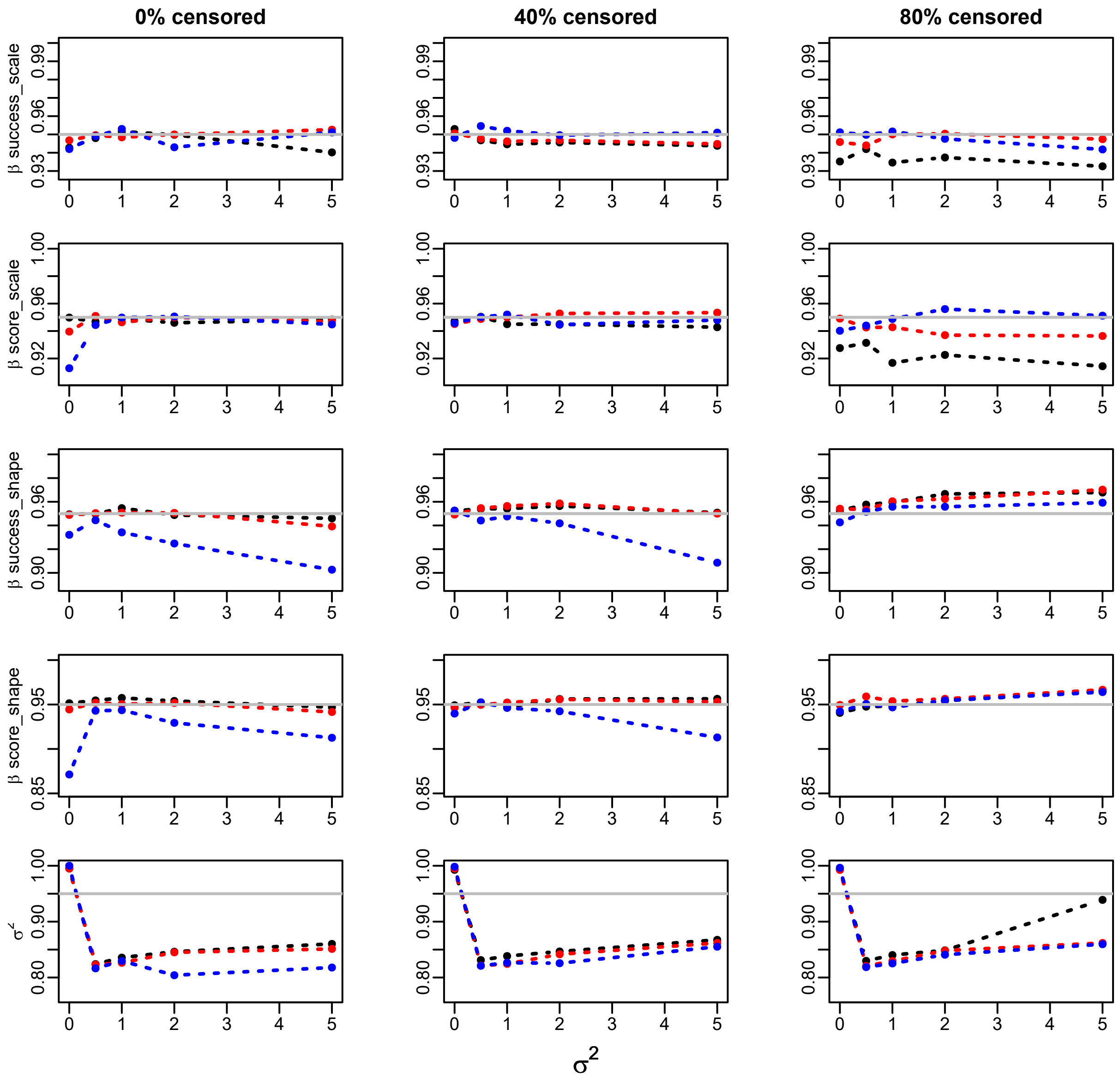

Figure A.11: Coverage of the standard errors based confidence intervals for the Cox regression parameters and σ^2 at nominal 95% level. Weibull model. Success proportion= 0.25 . 10 clusters. Sample sizes: 300 (black), 1000 (red) and 10000 (blue). True values: $\beta_{success-scale} = 0.5$, $\beta_{success-shape} = 0.05$, $\beta_{score-scale} = 1$, $\beta_{score-shape} = 0.1$

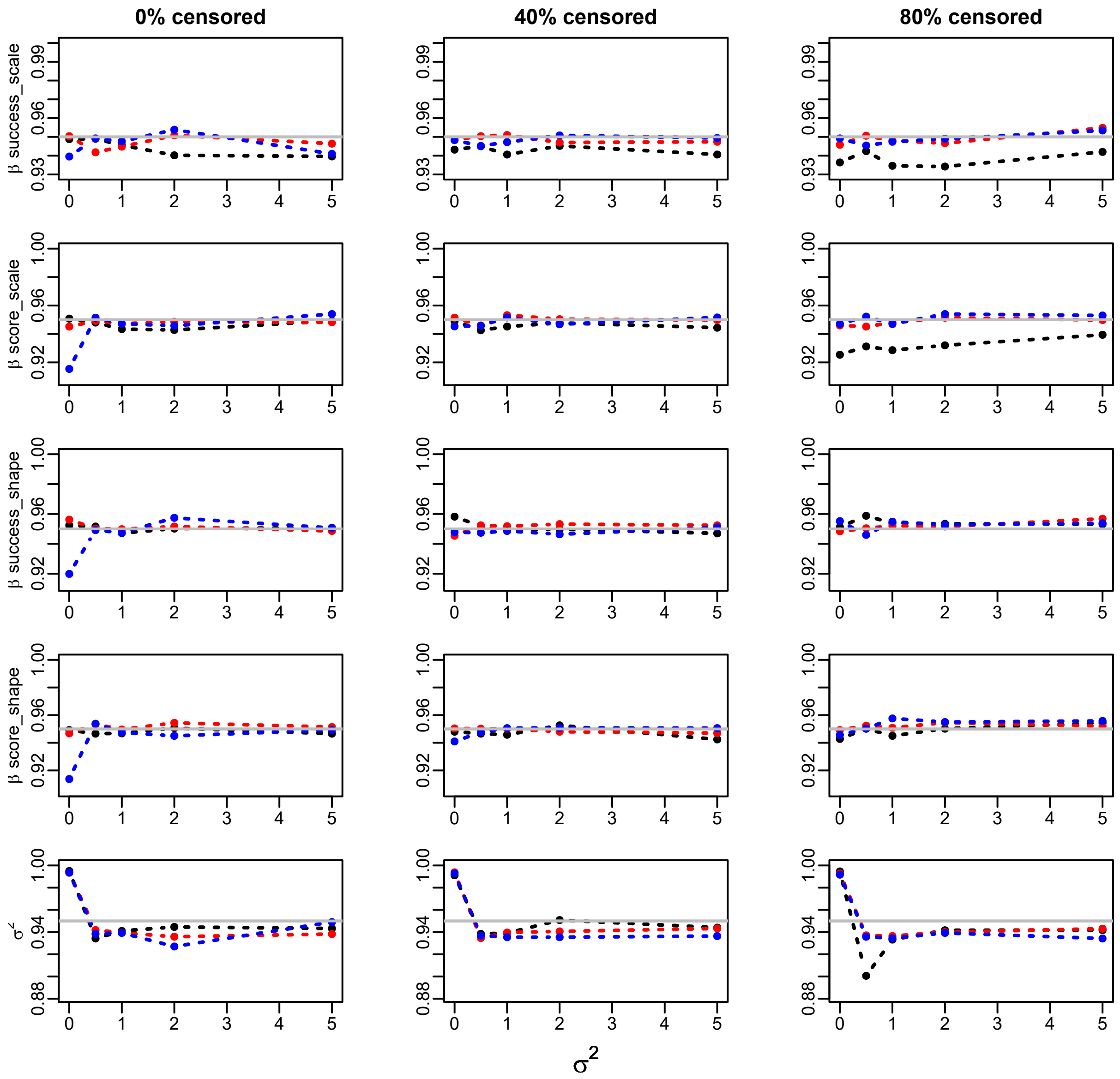

Figure A.12: Coverage of the standard errors based confidence intervals for the Cox regression parameters and σ^2 at nominal 95% level. Weibull model. Success proportion= 0.25. 100 clusters. Sample sizes: 300 (black), 1000 (red) and 10000 (blue). True values: $\beta_{success-scale}$ = 0.5 , $\beta_{success-shape}$= 0.05 , $\beta_{score-scale}$= 1 , $\beta_{score-shape}$= 0.1

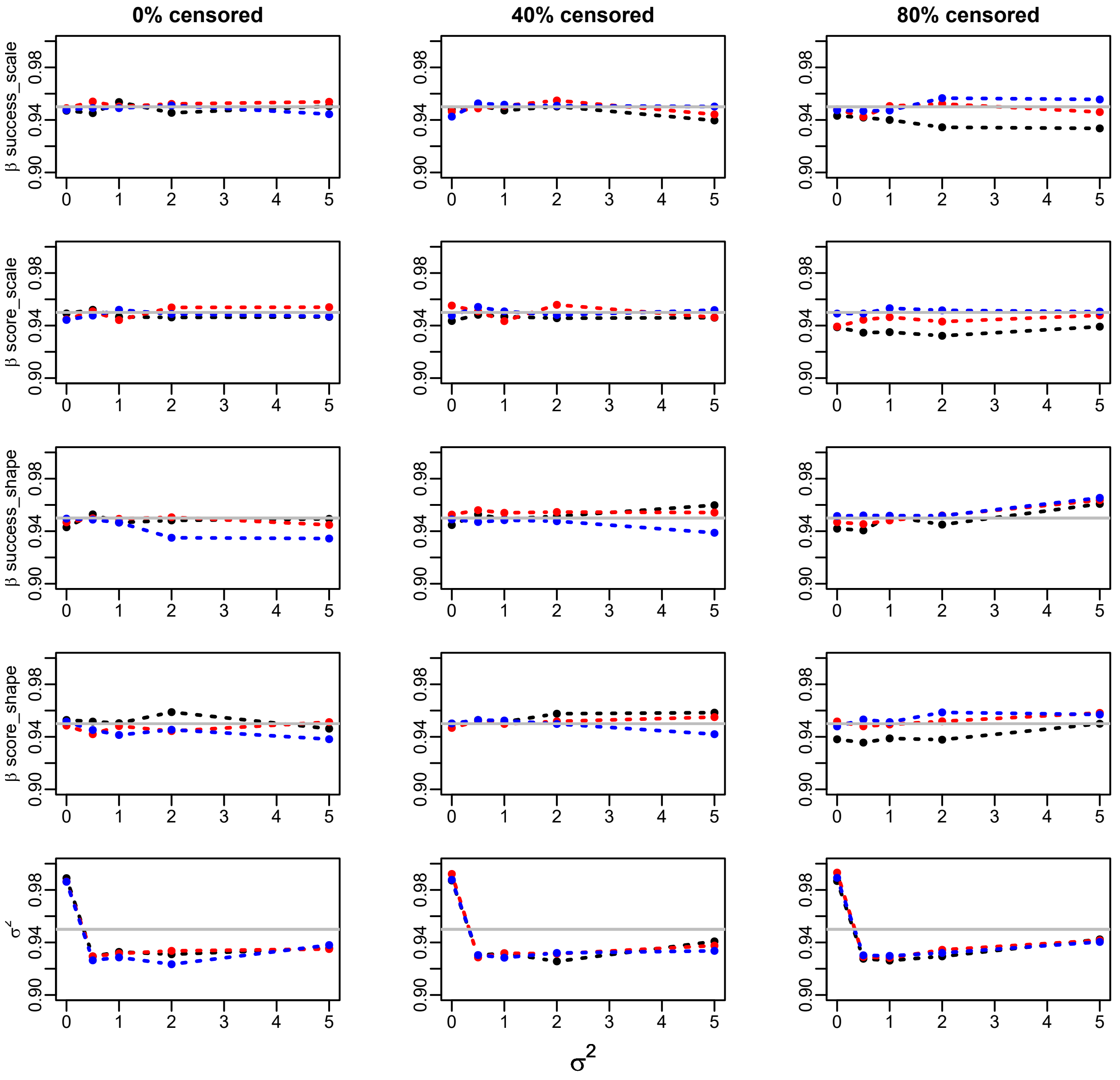

Figure A.13: Coverage of the profile likelihood confidence intervals for the Cox regression parameters and σ^2 at nominal 95% level. Weibull model. Success proportion= 0.25. 10 clusters. Sample sizes: 300 (black), 1000 (red) and 10000 (blue). True values: $\beta_{success-scale}$ = 0.5 , $\beta_{success-shape}$= 0.05 , $\beta_{score-scale}$= 1 , $\beta_{score-shape}$= 0.1

A.4.3 Coverage of the SE and the PL confidence intervals for the Gompertz model

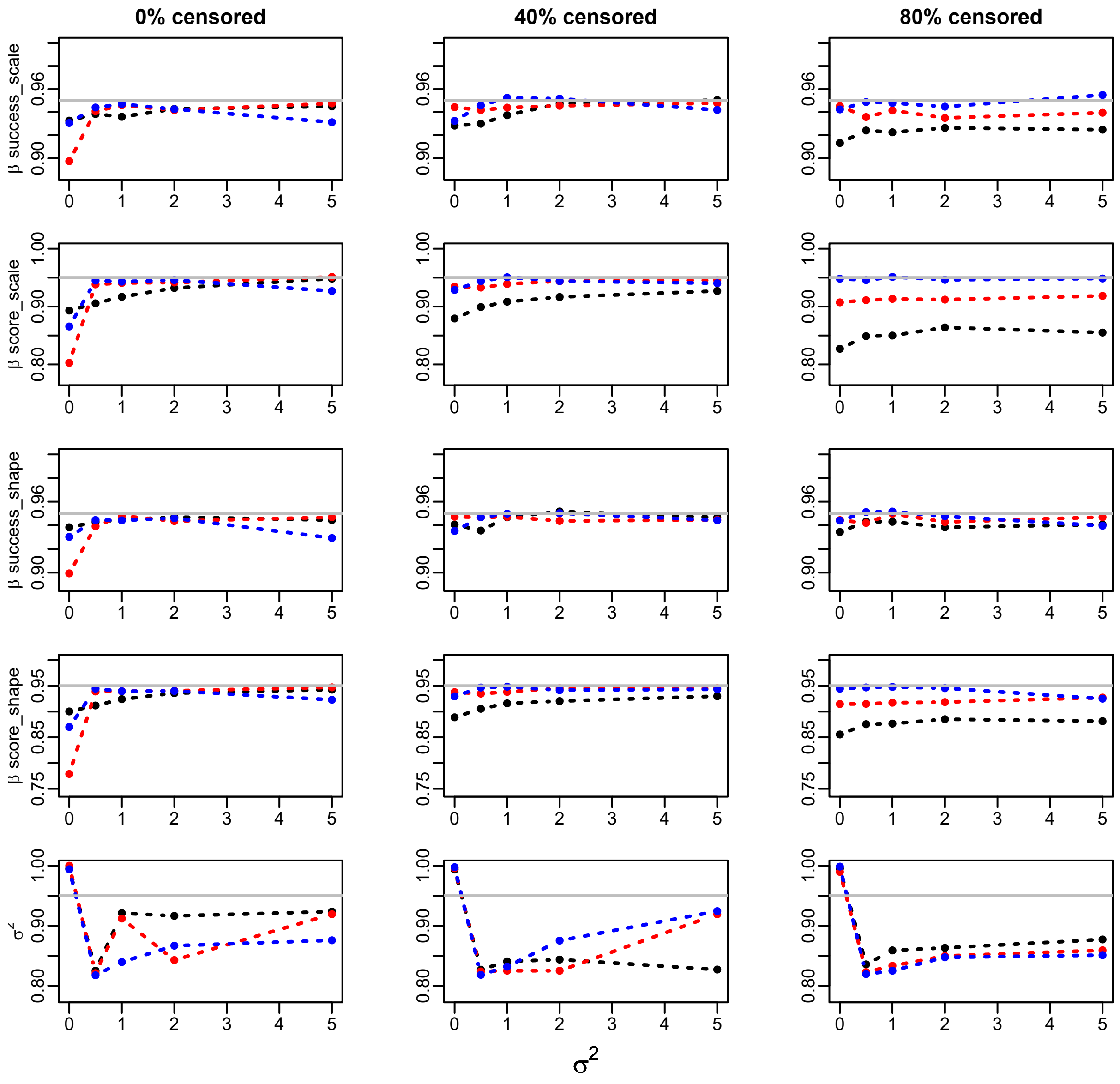

Figure A.14: Coverage of the standard errors based confidence intervals for the Cox regression parameters and σ^2 at nominal 95% level. Gompertz model. Success proportion= 0.25 . 10 clusters. Sample sizes: 300 (black), 1000 (red) and 10000 (blue). True values: $\beta_{success-scale}$ = -0.5 , $\beta_{success-shape}$= 0.05 , $\beta_{score-scale}$= -1 , $\beta_{score-shape}$= 0.1

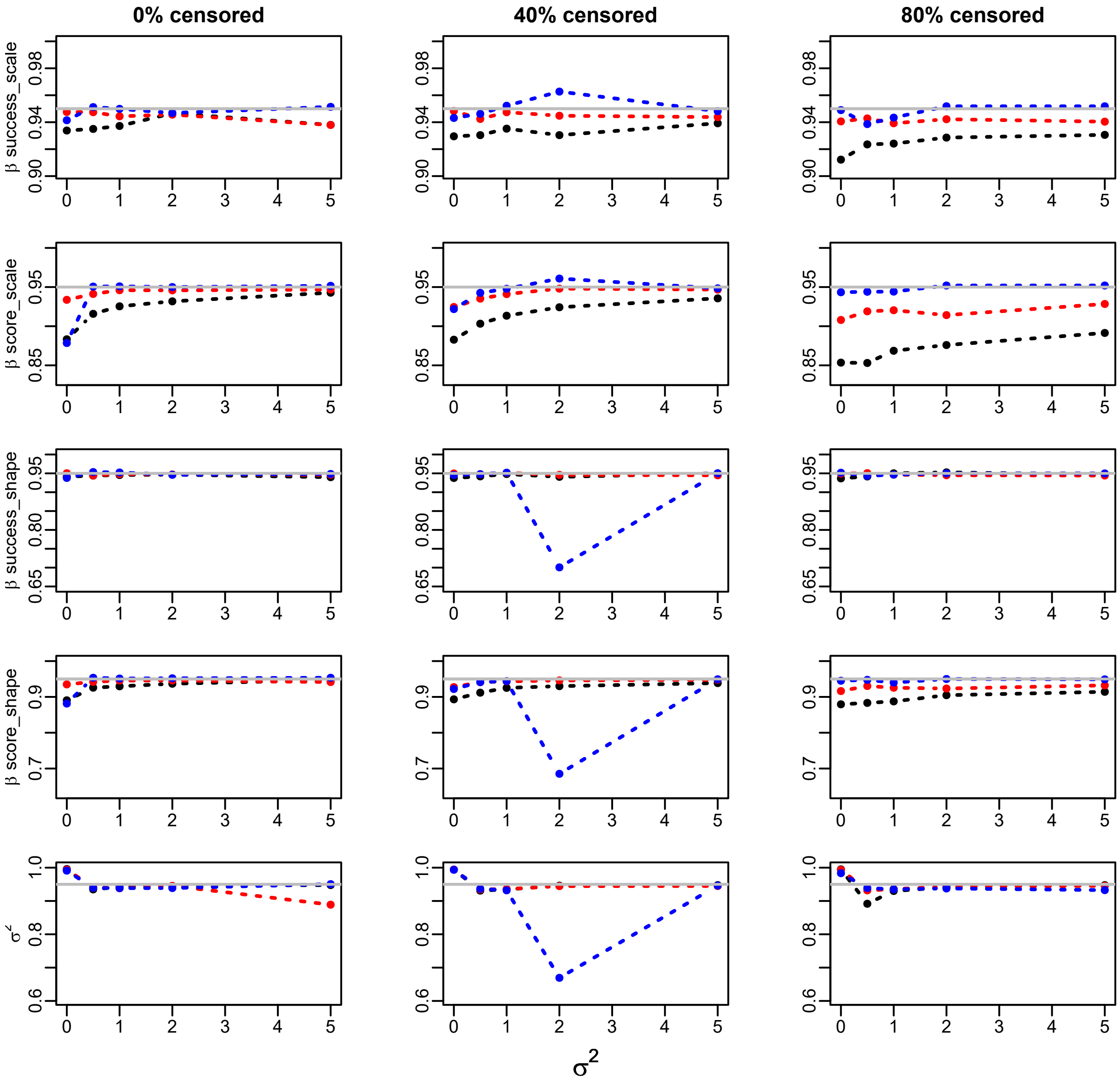

Figure A.15: Coverage of the standard errors based confidence intervals for the Cox regression parameters and σ^2 at nominal 95% level. Gompertz model. Success proportion= 0.25. 100 clusters. Sample sizes: 300 (black), 1000 (red) and 10000 (blue). True values: $\beta_{success-scale}$ = -0.5 , $\beta_{success-shape}$= 0.05 , $\beta_{score-scale}$=-1 , $\beta_{score-shape}$= 0.1

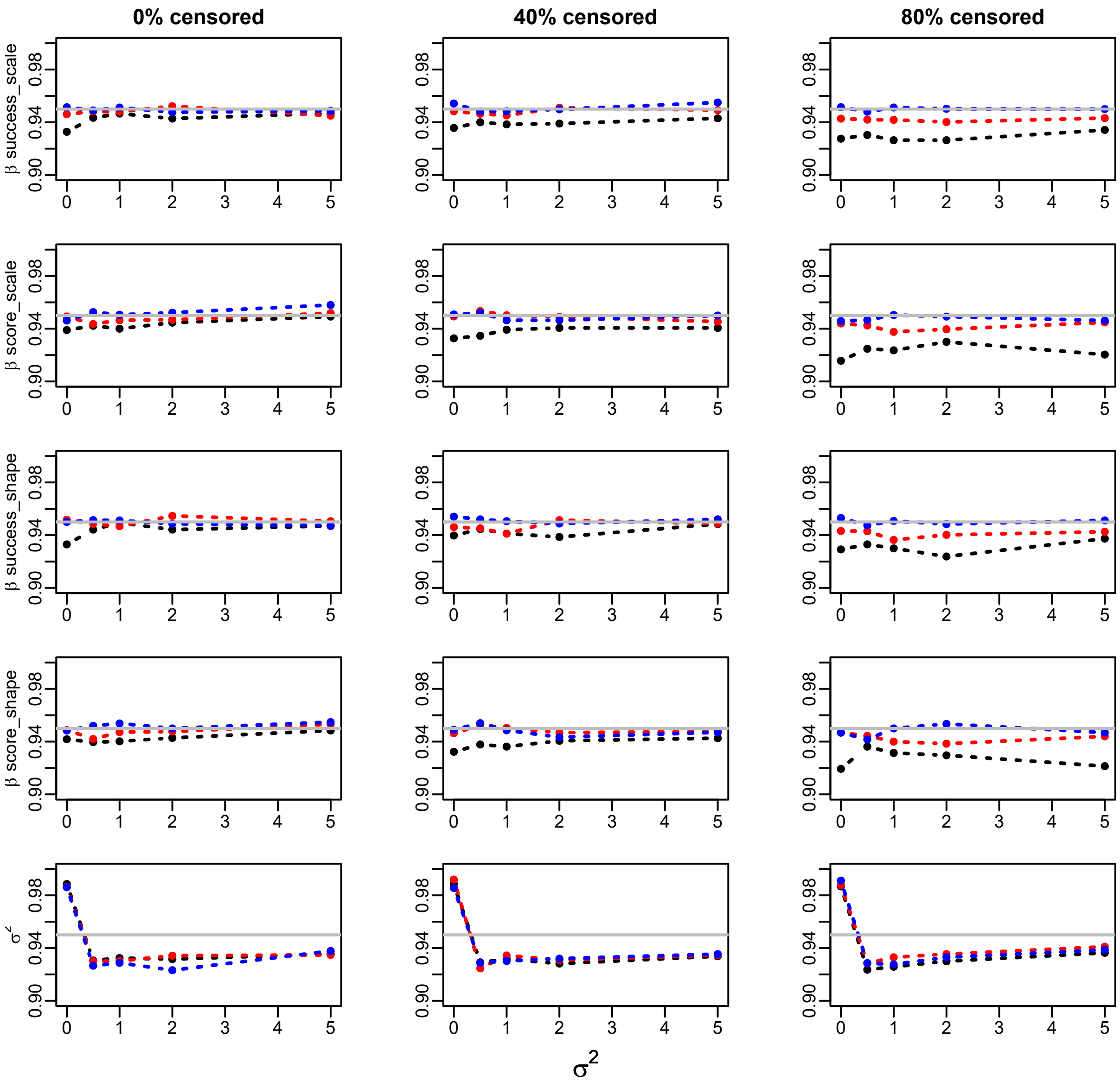

Figure A.16: Coverage of the profile likelihood confidence intervals for the Cox regression parameters and σ^2 at nominal 95% level. Gompertz model. Success proportion= 0.25 . 10 clusters. Sample sizes: 300 (black), 1000 (red) and 10000 (blue). True values: $\beta_{success-scale} = 0.5$, $\beta_{success-shape}= 0.05$, $\beta_{score-scale}= 1$, $\beta_{score-shape}= 0.1$

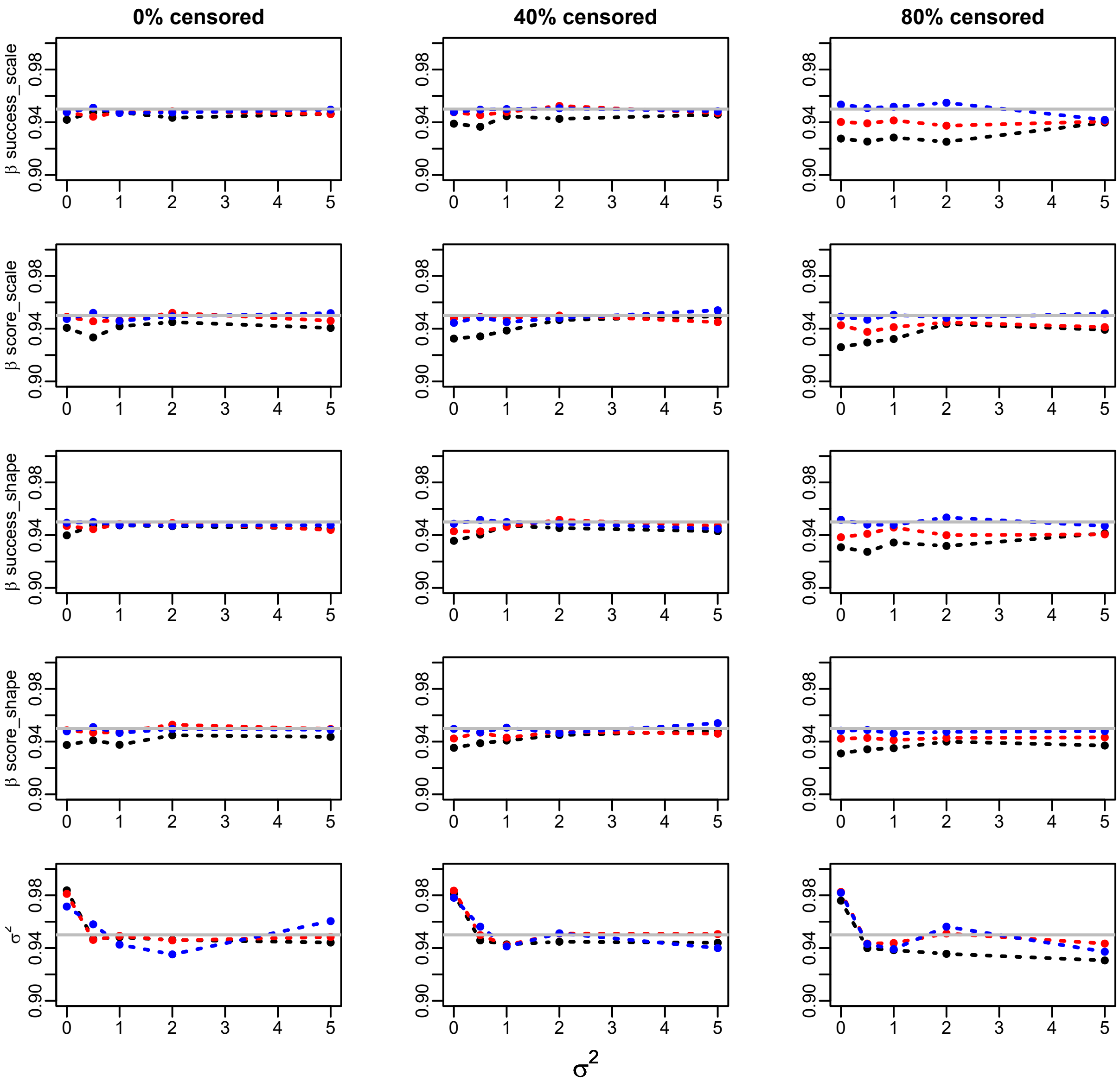

Figure A.17: Coverage of the profile likelihood confidence intervals for the Cox regression parameters and σ^2 at nominal 95% level. Gompertz model. Success proportion= 0.25. 100 clusters. Sample sizes: 300 (black), 1000 (red) and 10000 (blue). True values: $\beta_{success-scale} = 0.5$, $\beta_{success-shape}= 0.05$, $\beta_{score-scale}= 1$, $\beta_{score-shape}= 0.1$

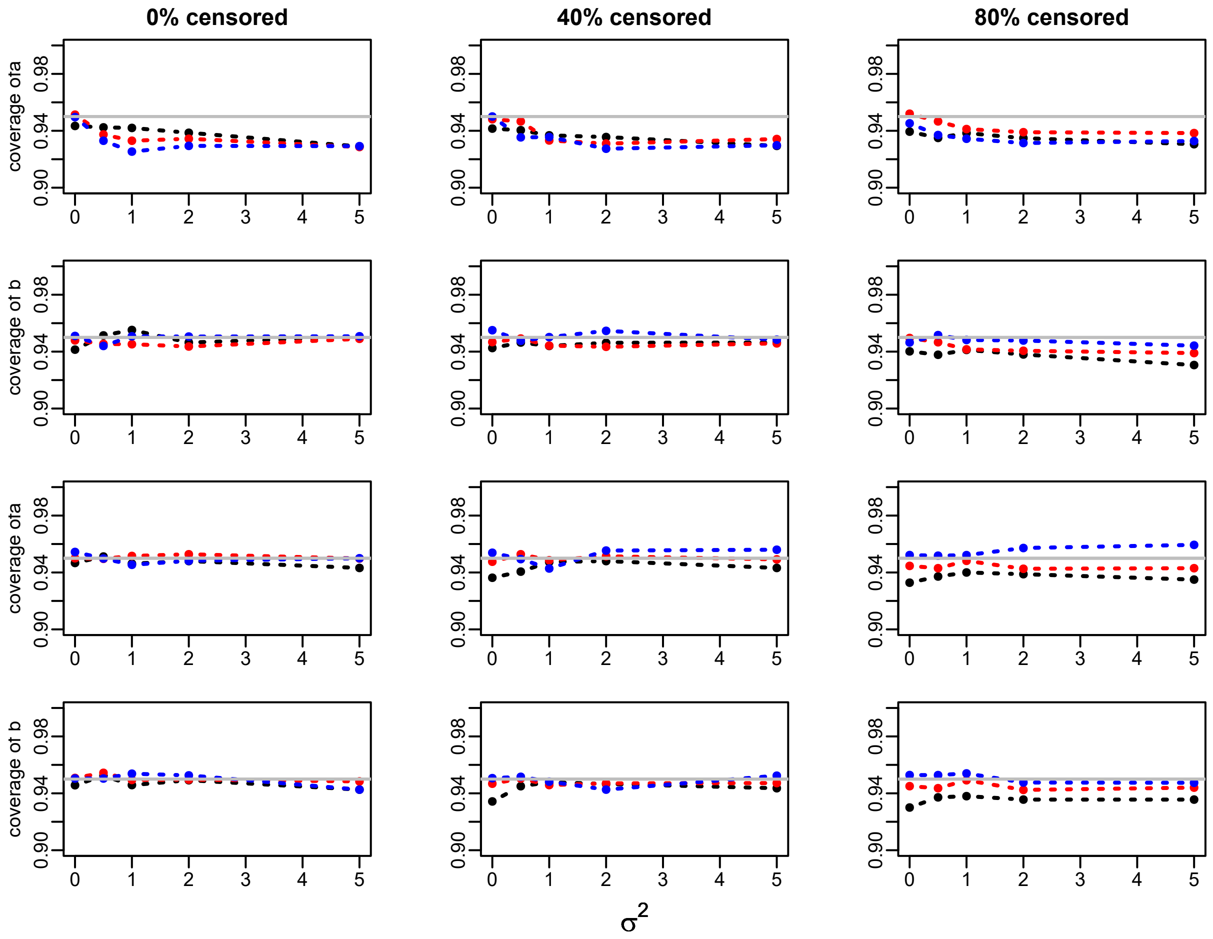

Figure A.18: Coverage of the profile likelihood confidence intervals for a and b parameters at nominal 95% level. Gompertz model. Success proportion= 0.25 . Sample sizes: 300 (black), 1000 (red) and 10000 (blue). True values: $\beta_{success-scale}$ = 0.5 , $\beta_{success-shape}$= 0.05 , $\beta_{score-scale}$= 1 , $\beta_{score-shape}$= 0.1. Top two rows: 10 clusters; bottom two rows: 100 clusters.

A.5 Assessment of the proportionality assumption in the standard Cox model of survival after a diagnosis of T2DM.

Table A5.1: **Cox PH model of survival after a diagnosis of T2DM and assessment of the proportionality assumption.** cox.zph procedure from *survival* R package estimates Pearson correlation between the scaled Schoenfeld residuals and the Kaplan-Meier estimators for each covariate. A p-value<0.05 indicates a violation of the proportionality assumption.

**Variable**	**Value**	**HR**	**95% CI**		**p-value**	**p-value (zph)**
Age at Diagnosis	Control	1				
	T2DM at 50-59	1.1836	1.1005	1.273	5.62E-06	0.069
	T2DM at 60-74	1.5041	1.4315	1.5804	<2e-16	
Birth Year	1930-1939	1				
	1940-1949	0.5763	0.5525	0.6011	<2e-16	<2e-16
	1950-1960	0.4193	0.3929	0.4475	<2e-16	
Gender	Female	1				0.954
	Male	1.372	1.3388	1.4061	<2e-16	
Smoking Status	Never	1				
	Former	1.6677	1.5649	1.7772	<2e-16	1.80E-05
	Smoker	2.6016	2.458	2.7535	<2e-16	
Deprivation (Townsend)	1	0.8421	0.8133	0.8721	<2e-16	
	2	0.9216	0.8901	0.9542	4.15E-06	
	3	1				0.166
	4	1.076	1.0385	1.1149	5.24E-05	
	5	1.2027	1.1573	1.25	<2e-16	
AF	YES	0.9967	0.9603	1.0346	0.86399	<2e-16
HF	YES	1.2162	1.1684	1.266	<2e-16	0.214

Continued on next page

Table A5.1 – continued from previous page

Variable	Value	HR	95% CI		p-value	p-value (zph)
MI	YES	1.3867	1.3267	1.4494	<2e-16	0.038
PVD	YES	1.0976	1.0611	1.1353	6.68E-08	0.89
HCL	None	1				
	Treated	0.9859	0.9542	1.0186	0.39315	3.70E-08
	Untreated	1.4215	1.3448	1.5024	<2e-16	
HTN	None	1				
	Treated	0.9446	0.9186	0.9714	6.34E-05	<2e-16
	Untreated	1.427	1.3836	1.4717	<2e-16	
BMI	Normal	1				
	Overweight	1.0056	0.9599	1.0535	0.81383	0.993
	Obese	1.1672	1.1082	1.2293	5.14E-09	
Age at Diagnosis	T2DM at 50-59:Former	0.7889	0.7058	0.8819	3.01E-05	
and Smoking Status	T2DM at 60-74:Former	0.7493	0.6978	0.8046	1.94E-15	0.149
	T2DM at 50-59:Smoker	0.3834	0.3416	0.4302	<2e-16	
	T2DM at 60-74:Smoker	0.4674	0.4313	0.5065	<2e-16	
Age at Diagnosis	T2DM at 50-59:Yes	0.6897	0.6027	0.7892	6.61E-08	0.014
and MI	T2DM at 60-74:Yes	0.7203	0.6688	0.7757	<2e-16	
Smoking Status	Former:Overweight	0.8731	0.8123	0.9385	0.00023	
and BMI	Smoker:Overweight	0.8067	0.7562	0.8606	7.71E-11	0.494
	Former:Obese	0.8457	0.7811	0.9156	3.51E-05	
	Smoker:Obese	0.8183	0.7583	0.8831	2.52E-07	
Birth Year	1950-1960:Former	0.9757	0.8803	1.0815	0.63966	<2e-16
and Smoking Status	1940-1949:Former	1.0563	0.9918	1.1249	0.08819	
	1950-1960:Smoker	1.2	1.0983	1.3111	5.42E-05	
	1940-1949:Smoker	1.1524	1.0845	1.2246	4.67E-06	
GLOBAL						<2e-16

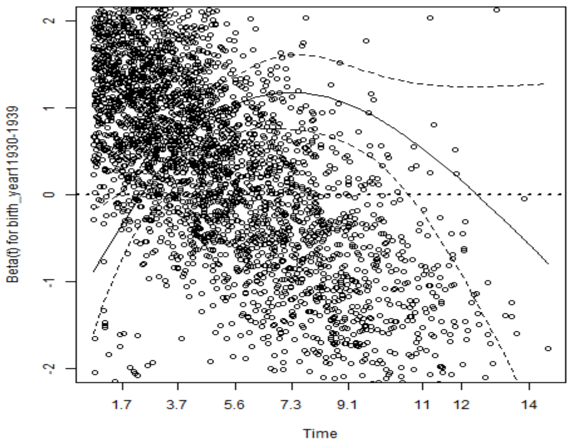

(a) Birth Cohort 1930-1939

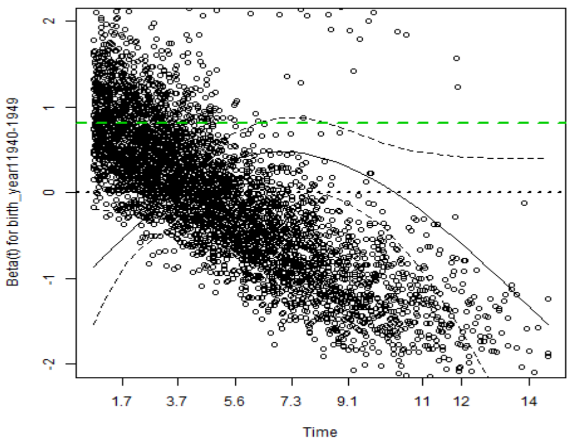

(b) Birth Cohort 1940-1949

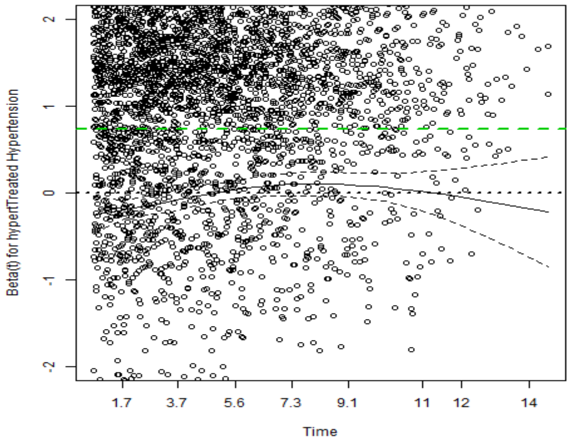

(c) Treated Hypertension

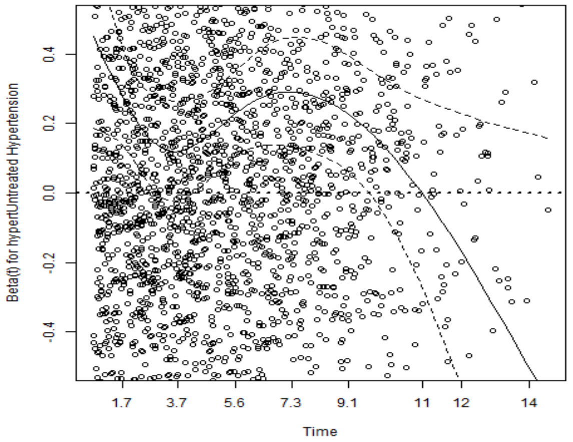

(d) Untreated Hypertension

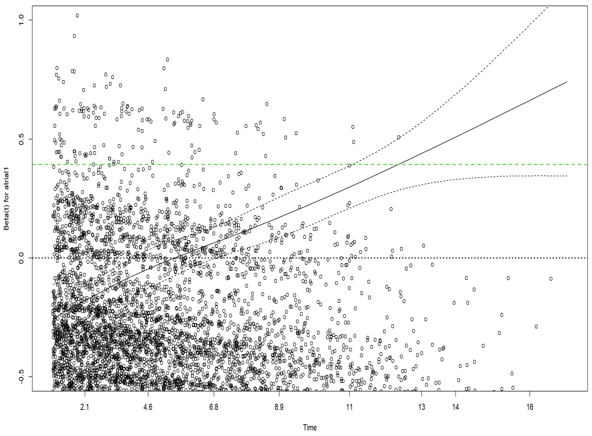

(e) Atrial Fibrillation

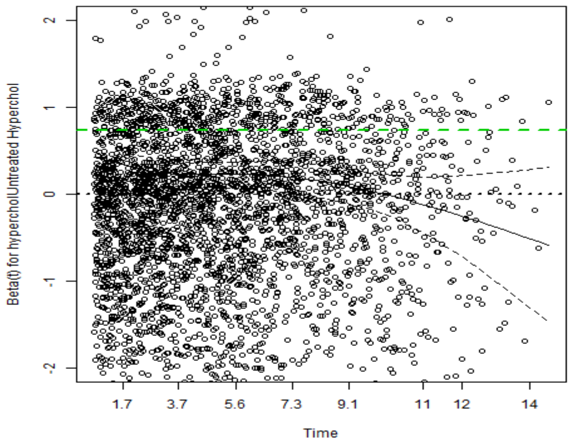

(f) Untreated Hypercholesterolemia

Figure A5.1: Plots of scaled Schoenfeld residuals demonstrating violation of the proportional hazards assumptions for birth cohort, hypertension (HTN), atrial fibrillation(AF) and untreated hypercholesterolemia (HCL).
